# Characterization of a two-component kinase that initiates the bacterial catabolism of hydroxyphenylethanones

**DOI:** 10.1016/j.jbc.2025.110210

**Published:** 2025-05-08

**Authors:** Gara N. Dexter, Jason C. Grigg, Michael Zahn, Eloisa J. Wheatley, Jennifer Lian, William W. Mohn, Lindsay D. Eltis

**Affiliations:** 1 Department of Microbiology and Immunology, Life Sciences Institute and Bioproducts Institute, The University of British Columbia, Vancouver, Canada; 2 Centre for Enzyme Innovation, School of the Environment and Life Sciences, University of Portsmouth, Portsmouth, UK

**Keywords:** phosphorylation, lignin valorization, kinase, biocatalysis, acetovanillone, bacterial metabolism, aromatic catabolism

## Abstract

The prodigious ability of bacteria to catabolize aromatic compounds has sparked considerable efforts to engineer bacteria to valorize lignin, an underutilized component of biomass. Despite decades of study, key catabolic pathways and enzymes remain poorly characterized. We recently identified the hydroxyphenylethanone (Hpe) pathway, which enables *Rhodococcus rhodochrous* GD02 and other bacteria to catabolize 4-hydroxyacetophenone (HAP) and acetovanillone (AV), which are generated in the catalytic fractionation of lignin. Catabolism is initiated by a two-component, ATP-dependent dikinase, HpeHI, homologs of which are involved in the catabolism of other aromatic compounds. In biochemical studies, the kinase activity of HpeHI was highest at low ionic strength and low concentrations of Mn^2+^. HpeHI had the highest apparent specificity for HAP and AV (*k*_cat_/*K*_M_ ≥ 250 mM^−1^ s^−1^) and had submicromolar *K*_M_ values for these substrates, consistent with the enzyme acting as a scavenging system. The enzyme also transformed 4-hydroxybenzaldehyde, vanillin, acetosyringone, and phenol. A 1.8 Å crystal structure of HpeI revealed that it is homologous to the ATP-grasp domain of rifampin phosphotransferase (RPH), while an AlphaFold model of HpeH indicated that it is homologous to the swivel and rifampin-binding domains of RPH. Consistent with HpeHI using a similar mechanism where the swivel domain transits between the spatially distinct substrate-binding sites, substitution of the conserved His residue in HpeH abolished kinase activity. Moreover, the HpeH component alone catalyzed phosphotransfer from 4-phosphoacetophenone to AV. This study reveals a subfamily of small-molecule dikinases that comprises two components, some of which are involved in aromatic compound catabolism.

Bacteria have evolved an exceptional ability to catabolize the manifold aromatic compounds found in the biosphere (1). This capability is essential to maintaining the global carbon cycle and has also been exploited for a range of biotechnological applications, from bioremediation to biocatalysis. One application that has gained recent attention is the valorization of lignin, a heterogeneous and recalcitrant aromatic polymer that comprises up to 30% of plant biomass (2, 3). Due to its abundance, lignin is an attractive feedstock to displace fossil fuels for the sustainable manufacturing of chemicals such as dicarboxylic acids and aromatics (4). One particularly promising emergent strategy for lignin valorization involves tandem processes that integrate chemical and biological catalysis (5, 6, 7). In these processes, thermochemical fractionation of the biomass yields a mixture of lignin-derived aromatic compounds (LDACs), which is then transformed by a microbial cell factory into a single, target compound. However, efficient production of the target compound requires that the biocatalyst be tailored to the mixture of LDACs, whose composition depends on the biomass and the chemo-catalytic depolymerization process (5). The tailoring of microbial cell factories requires that the requisite catabolic pathways and enzymes are well-characterized.

We recently described the previously unrecognized Hpe pathway, responsible for the catabolism of hydroxyphenylethanones such as acetovanillone (AV) and 4-hydroxyacetophenone (HAP) (8). Briefly, we isolated *Rhodococcus rhodochrous* GD02 for its ability to grow on AV, an LDAC that occurs in some industrial lignin streams, including black liquor (9). Genomic and biochemical studies revealed that the pathway comprises six enzymes (8). A homologous pathway enables the growth of *Sphingobium lignivorans* SYK-6 on AV and acetosyringone (10). Catabolism in GD02 is initiated by a two-component kinase, HpeHI, which catalyzes the ATP-dependent phosphorylation of AV or HAP to 4-phospho-AV or 4-phosphoacetophenone (PAP), respectively (Fig. 1) (8). The phosphorylated compounds are then carboxylated, dephosphorylated, and subjected to β-elimination to yield vanillate or 4-hydroxybenzoate from AV or HAP, respectively.

**Figure 1:**
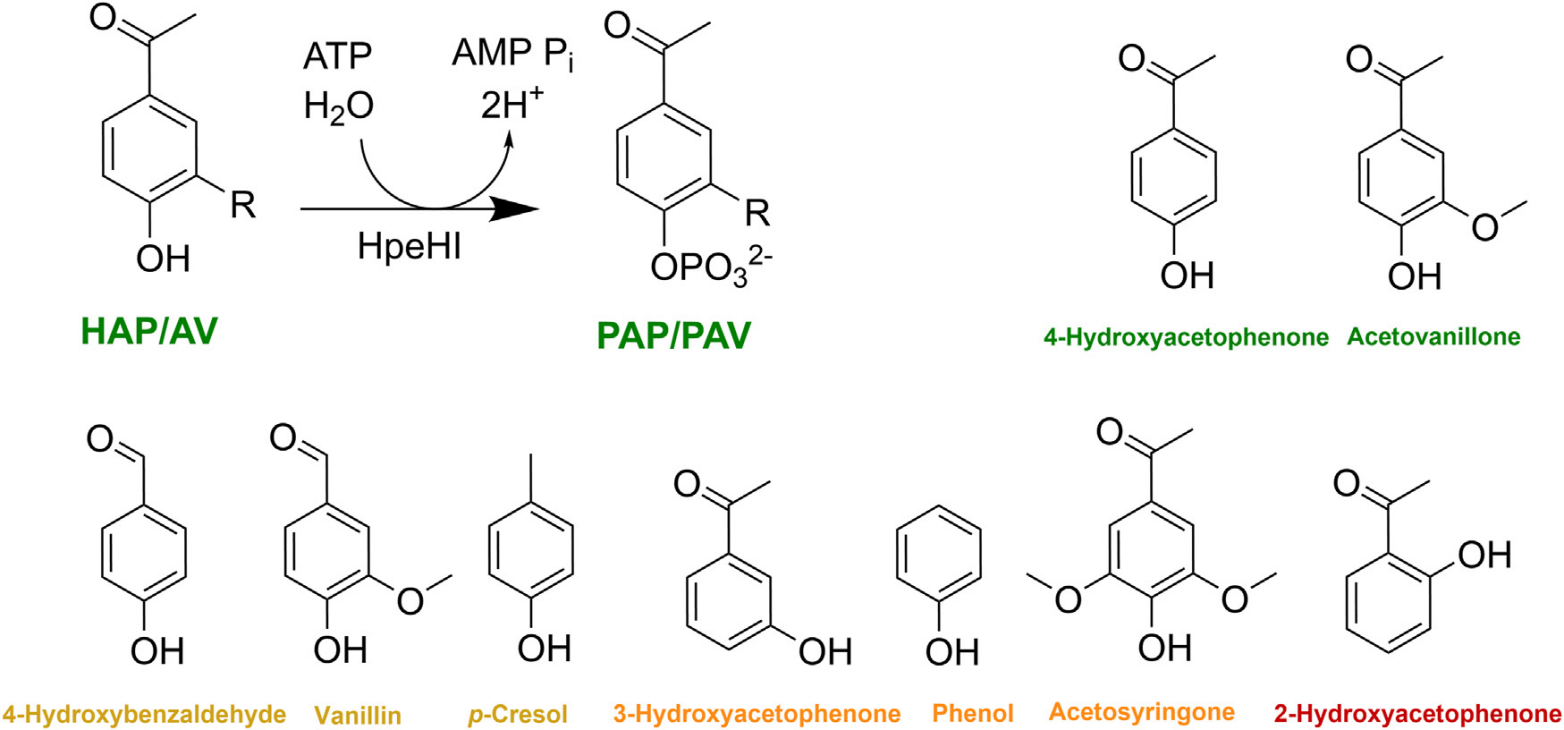
**HpeHI catalyzes phosphorylation of AV and HAP.** HpeHI is composed of two components: HpeI binds ATP and HpeH catalyzes the transfer of the β-phosphoryl group from ATP to the hydroxy group on the aromatic ring of 4-hydroxyacetphenone (HAP, R = H) or acetovanillone (AV, R=OCH_3_) to produce 4-phospho-acetophenone (PAP) or 4-phospho-acetovanillone (PAV), respectively. Analogs that were investigated as substrates are also depicted.

Although kinases involved in aromatic compound catabolism have not been well-characterized, several homologs of HpeHI have been reported to catalyze the phosphorylation of phenolic substrates. Among these, 4-methylbenzyl phosphate synthase, CreHI, initiates the catabolism of 4-cresol in *Corynebacterium glutamicum* (11). Similarly, phenylphosphate synthase, PpsAB, initiates the anaerobic catabolism of phenol in *Thaurea aromatica* (12), where it was originally identified as E_1_, and in *Aromatoleum aromaticum* strain EbN1^T^ (13). In HpeHI, CreHI, and PpsAB, both components are required for the ATP-dependent phosphorylation of the aromatic substrate (8, 11, 12). Moreover, PpsAB acts as a dikinase (12). Indeed, there is homology between PpsA and the central domain of phosphoenolpyruvate (PEP) synthase. For its part, PEP synthase is homologous to pyruvate phosphate dikinase (PPDK) and at least some of its domains also share homology with enzyme I of the PEP:sugar phosphotransferase system (PTS) and another dikinase, rifampin (RIF) phosphotransferase (RPH) (14, 15, 16). RPH deactivates RIF through ATP-dependent phosphorylation and is composed of three domains: an ATP-grasp domain, a RIF-binding domain, and a swivel domain. In the proposed mechanism of these enzymes, the swivel domain transits between the spatially distinct substrate-binding domains to transfer the phospho group from ATP to a second substrate *via* a conserved His residue (14, 15, 16). Phylogenetic analyses have revealed that RPH is part of a widespread family of small-molecule dikinases, with at least some members functioning on antibiotics (15). Further studies are needed to fully understand the evolutionary adaptations and substrate specificities of these enzymes.

In this study, we characterize HpeHI, the two-component kinase that initiates the catabolism of AV and HAP in *R. rhodochrous* GD02 (8). We first optimized reaction conditions with respect to pH, ionic strength, and divalent cation concentrations. A spectrophotometric assay was developed that enabled continuous monitoring of the reaction and facilitated steady-state kinetic analyses of the enzyme’s specificity (*k*_cat_/*K*_M_) for AV, HAP, 4-hydroxybenzaldehyde, vanillin, and ATP. In addition, the specific activity of HpeHI for further analogs of AV and HAP was determined. Structural analyses of the HpeI and HpeH components facilitated comparison with homologous kinases. Key active site residues were substituted using oligonucleotide-directed mutagenesis to probe their catalytic roles. The results are discussed with respect to the catalytic mechanisms of HpeHI and its homologs as well as the catabolism of aromatic compounds.

## Results

### Optimizing conditions for HpeHI activity

The two components of the kinase, HpeH and HpeI, were produced separately in *Escherichia coli*. Analysis on denaturing gels revealed that the protein preparations were >95% homogeneous and that the tagged and cleaved proteins matched their predicted sizes: 71.1 kDa for HpeH and 40.6 kDa for HpeI (Fig. S1). The CreHI and phenylphosphate synthase activities were evaluated using discontinuous assays based on radioautography or HPLC (11, 12). To facilitate the characterization of these systems, we developed a continuous assay for HpeHI based on the differences in the absorption spectra of the substrate and product (see Supplementary Methods). The extinction coefficients for substrates were as follows: Δε_325nm_ for HAP and PAP was 6.2 mM^−1^ cm^−1^, Δε_314_ for AV and PAV was 2.85 mM^−1^ cm^−1^, Δε_330_ for 4-hydroxybenzaldehyde was 11.6 mM^−1^ cm^−1^, and Δε_340nm_ for vanillin was 12.4 mM^−1^ cm^−1^.

We next used the continuous assay to evaluate the influence of buffer conditions on the specific activity of HpeHI for HAP. Specifically, we investigated the effect of pH, ionic strength, and divalent cations, basing the specific activity on the amount of HpeH. Using buffer conditions similar to those for HpeHI homologs (11, 12), the specific activity of the enzyme was approximately 50% higher at pH 8.5 than 7.5 (Fig. S3). Further experiments were performed at pH 7.5 as this is closer to physiological conditions. At this pH, the activity of HpeHI decreased with increasing ionic strength, *I* (Fig. S4). Thus, at the highest ionic strength tested, 480 mM, the specific activity of the enzyme was less than half of what it was at the lowest ionic strength tested, 18 mM.

**Figure 2:**
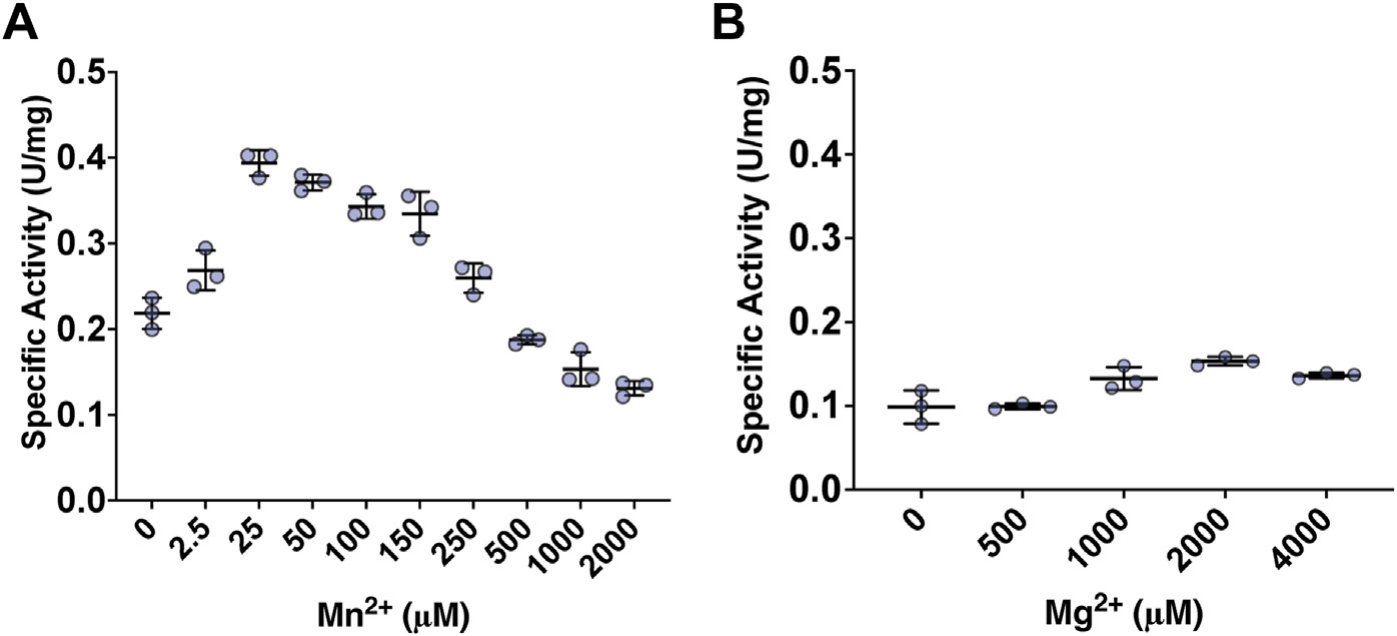
**Dependence of HpeHI activity on divalent cations.***A*, specific activity of HpeHI with varying Mn^2+^ and 2.0 mM Mg^2+^ concentrations. *B*, specific HpeHI activity with varying Mg^2+^ and 1.0 mM Mn^2+^ concentrations. Activities were measured using the spectrophotometric assay. Filled circles represent individual replicates. Error bars represent the standard deviation. Statistical significance was assessed using an unpaired *t* test to compare each treatment to the 0 mM (no added Mn^2+^ or Mg^2+^) control. For panel (*A*), from left to right, *p*-values are: 0.043, 0.0002, 0.0002, 0.001, 0.003, 0.047, 0.048, 0.014, 0.002, and for panel (*B*), *p*-values are: 0.964, 0.071, 0.010, and 0.032.

The activities of homologous aromatic kinases have been evaluated in the presence of 20 mM MgCl_2_ and 1.0 mM MnCl_2_ (11, 12). As these concentrations are relatively high, we sought to optimize them by evaluating the dependency of HpeHI activity on divalent cations. In these experiments, we maintained the ionic strength of the assay buffer at 29 mM using NaCl. Importantly, purified HpeH and HpeI had no detectable activity above background in the absence of exogenously added divalent cation. At 2.0 mM MgCl_2_, the specific activity of HpeHI was strongly dependent on Mn^2+^ concentration, with the highest activity, 0.39 ± 0.02 U/mg, observed at 25 μM Mn^2+^ (Fig. 2*A*). Interestingly, the enzyme had significant activity in the absence of exogenously added Mn^2+^. Indeed, this activity was higher than that observed above 0.5 mM Mn^2+^, indicating that excess Mn^2+^ is inhibitory. At 1 mM MnCl_2_, the activity of HpeHI was maximal at 2.0 mM MgCl_2_ (Fig. 2*B*). Finally, at 1 mM MnCl_2_, the enzyme also had significant activity in the absence of exogenously added Mg^2+^.

The dependence of HpeHI activity on Mn^2+^ prompted us to investigate the effect of other divalent metal ions on the enzyme’s activity. Accordingly, we tested the ability of Co^2+^, Ni^2+^, and Zn^2+^ to substitute for Mn^2+^. For this experiment, 50 μM of the tested divalent metal ion was used in the presence of 2.0 mM MgCl_2_. In the presence of Co^2+^ and Ni^2+^, the activity of HpeHI was approximately 73% and 81%, respectively, of that observed with Mn^2+^ (Table 1). This was significantly more than the activity observed in the absence of Mn^2+^ (Fig. 2*A*), indicating that Co^2+^ and Ni^2+^ can partially compensate for the lack of Mn^2+^ and stimulate HpeHI activity. By contrast, the activity in the presence of Zn^2+^ was ∼13% of that observed with Mn^2+^. This was significantly less than the activity observed in the absence of added MnCl_2_, indicating that Zn^2+^ inhibits HpeHI activity.

**Table 1:** Activity of HpeHI with different divalent cations^a^

Cation	Specific activity
	U/mg HpeH
none	0.22 (0.02)
Mn^2+^	0.37 (0.01)
Ni^2+^	0.30 (0.04)
Co^2+^	0.27 (0.02)
Zn^2+^	0.05 (0.01)

a Assay buffer contained 2.0 mM MgCl_2_ and 50 μM of the indicated divalent metal ion. Standard deviation in parentheses for reactions performed in triplicate.

Based on the abovementioned studies, further kinetic studies of HpeHI were performed using an assay buffer of ionic strength (*I* = 20) containing 25 μM Mn^2+^ and 2.0 mM Mg^2+^. Using these conditions, we next investigated the effects of enzyme component stoichiometry on the reaction rate. In reactions containing 0.010 μM HpeH, the initial rate of HAP phosphorylation depended linearly on the concentration of HpeI up to at least 5.0 μM (Fig. S5*A*). Similarly, in reactions containing 1.0 μM HpeI, the rate depended linearly on the concentration of HpeH up to at least 0.10 μM (Fig. S5*B*). Based on these data, steady-state kinetics studies were conducted using 0.05 μM HpeH and 0.10 μM HpeI to maximize the observable rates while minimizing background. Finally, to test whether HpeHI is a dikinase, we analyzed reaction mixtures using LC-QTOF. Indeed, ATP levels were depleted, and AMP was the predominant product (Fig. S6). The low amounts of ADP detected are consistent with the activity of homologous dikinases.

### Steady-state kinetic parameters of HpeHI

Under standard assay conditions, the HpeHI reaction obeyed Michaelis-Menten kinetics with respect to 4-hydroxybenzaldehyde, vanillin, and ATP (Fig. S7). The *K*_M_ values for HAP and AV were too low to accurately determine Michaelis-Menten parameters using this assay. Accordingly, those values and substrate specificity (*k*_cat/_*K*_M_) are represented as upper- and lower-bounds, respectively. Importantly, initial reaction rates measured spectrophotometrically at low concentrations of AV and HAP were verified using an LC-MS-based assay that measured the amount of phosphorylated product. Steady-state kinetic parameters were based on the concentration of HpeH in the assay and are apparent, given the strong dependence of the rate on the concentration of HpeI. The apparent substrate specificity (*k*_cat/_*K*_M_) of HpeHI for HAP and AV were similar to each other and > 20-fold higher than for 4-hydroxybenzaldehyde and vanillin (Table 2). However, we note the submicromolar apparent *K*_M_ of the enzyme for both HAP and AV is likely much lower than reported and that the enzyme turned over the two substrates at similar apparent *k*_cat_ values. At saturating amounts of HAP, the apparent *K*_M_ of HpeHI for ATP was over two orders of magnitude higher than for either HAP or AV.

**Table 2:** Steady-state kinetics parameters^a^

Substrate	*k*_cat_	*K*_M_	*k*_cat_/*K*_M_
	s^−1^	μM	mM^−1^ s^−1^
Acetovanillone	0.07 (0.01)	<0.3 (0.1)	>250 (100)
4-hydroxyacetophenone	0.08 (0.01)	<0.3 (0.1)	>290 (100)
4-hydroxybenzaldehyde	0.11 (0.01)	9.6 (1.8)	11 (2)
Vanillin	0.093 (0.002)	10.3 (0.6)	9 (0.4)
ATP^b^	1.92 (0.02)	45 (3)	0.04 (0.00)

a Experiments were performed using 0.05 μM HpeH, 0.10 μM HpeI in a buffer (*I =* 20 mM) containing HEPES, 3.9 mM NaCl, 2.0 mM MgCl_2_, 25 μM MnCl_2_ and 0.50 mM TCEP at pH 7.5, 30 °C. Reactions with aromatic substrates contained 500 μM ATP. Parameters were calculated using LEONORA and a minimum of 15 data points at various substrate concentrations. Values of *k*_cat_ were calculated based on the concentration of HpeH. Standard deviation is shown in parentheses.

b Parameters were evaluated using 100 μM HAP.

### Substrate preference of HpeHI

To evaluate the ability of HpeHI to phosphorylate a broader range of phenolic compounds, we used an LC-MS assay. Moreover, we used higher concentrations of HpeH and HpeI, given the low activity of the enzyme for some of the tested substrates. Specific activities for AV, HAP, and phenol were based on quantitation of the phosphorylated products. Specific activities for the remaining substrates were based on the depletion of the phenolic substrate, as the corresponding phosphorylated products were not available as authentic standards. Under these conditions, HpeHI had the highest specific activity for HAP and AV (Table 3). HpeHI also had significant activity for 4-hydroxybenzaldehyde, vanillin, and cresol, turning the latter over at nearly a quarter of the rate of HAP. Acetosyringone, 3-hydroxyacetophenone, and phenol were slowly transformed as verified by the detection of phosphorylated products. Of the tested compounds, only 2-hydroxyacetophenone was not detectably transformed.

**Table 3:** Specific activities of HpeHI on aromatic substrates

Substrate	Specific activity
	U/mg HpeH
Acetovanillone	0.74 (0.01)
4-hydroxyacetophenone	0.69 (0.003)
4-hydroxybenzaldehyde	0.41 (0.02)
Vanillin	0.27 (0.03)
Cresol	0.18 (0.01)
3-hydroxyacetophenone	0.038 (0.001)
Phenol	0.038 (0.001)
Acetosyringone	0.025 (0.002)
2-hydroxyacetophenone	ND

The activity of HpeHI was measured using 100 μM of each phenolic substrate, 500 μM ATP (HEPES buffer (*I* = 20 mM), pH 7.5, 30 °C). Rates were based on substrate depletion except for HAP, AV, and phenol, for which specific activity was determined by monitoring product formation. Specific activities were based on the amount of HpeH in the reaction. The values represent the mean and standard deviations of three technical replicates. ND, no phosphorylated product detected.

### HpeH is capable of phosphate transfer

The PEP synthase from *T. aromatica* is capable of transferring phosphate from phenylphosphate to labelled substrate. To investigate whether HpeH alone was similarly capable of phosphate transfer from substrates other than ATP, transfer from PAP to AV was monitored using a discontinuous LC-MS-based assay. Reactions were quenched at 2, 10, and 30 min, and PAV was quantified using a standard curve. Indeed, HpeH alone was capable of phosphate transfer to AV at a rate of 2.7 ± 0.5 nmol min^−1^ mg^−1^ (Table S2). Adding the ATP analog, AMP-PNP, and/or HpeI did not significantly affect the rate of phosphate transfer.

### Structural characterization of HpeH and HpeI

**Figure 3:**
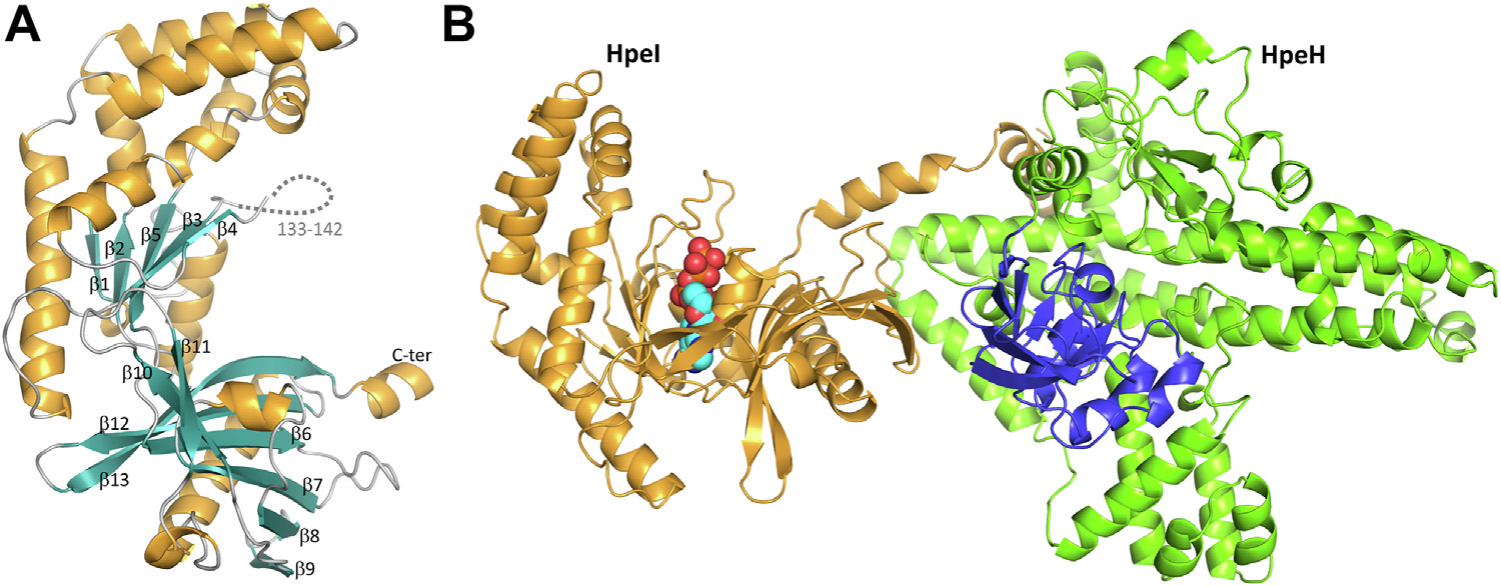
**X-ray crystal structure of HpeI and a model for the HpeHI-ATP complex.***A*, the X-ray crystal structure of HpeI is shown as a cartoon with helices, strands, and loops indicated in *orange*, *blue*, and *gray*, respectively. β-strands are numbered for clarity, and the unmodeled loop is indicated by a *grey* dashed line. *B*, An AlphaFold 3.0 model of the ternary complex between HpeH, HpeI, and ATP. HpeI is shown in *orange* with a molecule of ATP (*cyan*, *blue*, and *red*) modeled as spheres. HpeH is shown in *green* (substrate binding domain) and *blue* (His or swivel domain).

To understand the mechanism of substrate specificity in the HpeHI system, we sought to characterize the structures of both components. Crystals of HpeI diffracted to 1.8 Å resolution, and the structure was solved by molecular replacement using an HpeI AlphaFold model (AF-A0A520FAS4-F1-v4), with the N- and C-terminal lobes as separate search models. Data collection and refinement statistics are shown in Table S3. A PDB search utilizing the DALI protein structure comparison server identified the ATP-grasp domain of RPH (15, 16) as the closest structural homolog (PDB IDs: 5HV6, 5FBS) with RMSD values of 4.5 and 5.0 Å over 302 and 281 amino acids, respectively, followed by the ATP-grasp domain of PPDKs from *Clostridium symbiosum* and maize (PDB IDs: 2R82, 1VBG) with RMSD values of 2.3 and 3.1 Å over 235 and 237 residues, respectively (17, 18). The HpeI structure is comprised of two α/β lobes from residues 1 to 196 and 201 to 340, respectively, bridged by a single loop between β5 and β6 (Fig. 3*A*).

ATP-grasp domains are widespread components of many enzymes that use ATP as a co-substrate. The ATP-binding site is located at the interface between the N- and C-terminal α/β lobes. Tjaden *et al.* previously proposed two distinct nucleotide binding motifs that could discriminate the grasp domains of PEP synthases and PPDKs (19). The motif comprises β-strands 3 and 4 and their joining loop (V124-V150) located in the N-terminal α/β lobe. To understand the importance of these sequence motifs in HpeI and the broader implications for ATP-grasp domains, we generated structure-guided sequence alignments of the corresponding domains from the characterized phenolic substrate kinases, CreI and PpsB, as well as RPH, *E. coli* PEP synthase, and *C*. *symbiosum* PPDK (Fig. S8). The motif from all phenolic substrate kinases more closely resembles PEP synthase than PPDK. Notably, residues 133 to 142 comprising the conserved loop could not be modeled in our HpeI structure and are presumably disordered. The same loop was unmodeled in the ADP-bound structure of RPH (PDB ID: 5FBS), but the significantly shorter loop found in PPDK was modeled in the substrate-free structures mentioned above (PDB IDs: 2R83, 1VBG).

Another notable feature of the HpeI structure is that it is in a more open conformation than many characterized grasp domains (Fig. S9*A*). The ATP grasp domains from RPH and PPDK structures were shown to undergo a hinge-like ATP-dependent conformational change between the two α/β lobes to enclose ATP (15, 17, 19). Given that HpeI was crystallized in the absence of ATP, the crystals likely represent the open conformation, and it would similarly undergo ATP-dependent closure upon binding, though no crystals of the nucleotide-bound form were obtained.

Next, we sought to characterize the HpeH Substrate-binding and Swivel domains. We were unable to obtain crystals of the protein, so an AlphaFold DB model was generated and used for all subsequent structural analysis (Fig. S10). A DALI search using HpeH reveals no close homologs in the PDB. RPH is the only structure that aligns over most of the HpeH structure (RMSD of 3.6 Å and 20% sequence identity over 473 amino acids, PDB ID: 5FBT) (Fig. S9*B*). Several other homologs identified include PEP synthase and PPDK structures, but the alignment is limited to the C-terminal ∼120-residue Swivel domain. A structure-guided sequence alignment of HpeH with the characterized phenolic substrate kinases, CreH and PpsA, as well as RPH (PDB ID: 5HV1), PEP synthase (PDB ID: 2OLS), PPDK (PDB ID: 1DIK), and PtsI (PDB ID: 2HWG) was generated using T-Coffee Expresso using deposited structures (Fig. S11). HpeH, CreH, and PpsA all aligned with ∼45% pairwise identity, while RPH shares ∼20% sequence identity over the length of HpeH. PEP synthase, PPDK, and PtsI homology is limited to the Swivel domain (∼18–35% identity over 100–160 amino acids). Despite the divergence, conserved sequences were identified in the Swivel domains, particularly around the histidine responsible for phosphate transfer, H581 in HpeH. The sequence preceding His581 is conserved as T/SXXGGXXXH, appearing to be a key component of the Swivel domain, with the δO of T/S (S573 in HpeH) forming a hydrogen bond with the backbone carbonyl of the residue at +2, followed by the G-rich loop that orients the conserved His for phosphotransfer. Notably, a conserved Thr is present in PEP synthase, PPDK, and PtsI (position 579 in HpeH numbering) that is the site of regulatory phosphorylation. In HpeH and the other homologs examined, this residue is a methionine, suggesting the RPH and phenolic substrate kinases are not similarly regulated by phosphorylation.

The phosphate transfer from HpeI-bound ATP to HpeH-bound substrate is likely mediated by formation of an HpeHI complex that would resemble the mechanism for RPH (15, 16). HpeHI complex formation in solution, was investigated using size-exclusion chromatography coupled to a multi-angle light scattering detector in the buffer described for kinetic analysis, supplemented with 20 mM or 200 mM NaCl to mitigate nonspecific interaction with the Superdex resin. HpeH alone runs with a mass of 122 ± 3 kDa and 143 ± 11 kDa in higher and low ionic conditions, respectively (Fig. S11). HpeH has a theoretical mass of 71.1 kDa, suggesting HpeH could exist as a dimer in solution. HpeI alone ran with a calculated mass of 52 ± 13 kDa at low ionic strength. The expected mass is 40.6 kDa, consistent with HpeI forming a monomer in solution. There was no evidence of stable complex formation observed at either ionic strength or in the presence of the non-hydrolysable ATP analog, AMP-PNP, suggesting complex formation may be transient and require a substrate or ATP binding (Fig. S12). Complex formation in low-ionic strength conditions was further investigated using mass photometry. The mass photometry data for HpeH alone was consistent with it existing in solution as a mixture of monomer and dimer. Again, HpeH and HpeI did not display evidence for stable complex formation. (Fig. S13). The intriguing potential for HpeH forming a homodimer was investigated using AlphaFold, which consistently predicted an interface across the extended helical region of the protein on the opposite face to the Swivel domain (Fig. S14).

AlphaFold 3.0 was then used to predict a ternary HpeH:HpeI:ATP complex (Figs. 3*B* and S15). The result is a plausible model for the transient complex, where ATP bound in HpeI is in close proximity to engage the HpeH Swivel domain and phosphorylate H581, followed by movement of the Swivel domain back to HpeH for phosphorylation of the phenolic substrate. The homolog, RPH is a fusion protein, equivalent to HpeHI and the domains interact across a similar interface as the HpeH:HpeI:ATP AlphaFold structure, placing the swivel domain approximately the same distance from the ATP-grasp domain-bound ATP (Fig. S16). Notably, an HpeHI complex generated by AlphaFold or by superposition on RPH both result in complementary surface electrostatics with a positively charged patch in HpeI against a complementary negative patch on HpeH (Fig. S16). Such an interaction could explain the enhanced activities observed at lower NaCl concentrations (Fig. S4).

### Mutation of key residues in HpeHI affects activity

Sequence and structural alignments with characterized homologs such as CreH and RPH support the hypothesis that HpeH His581 is phosphorylated during phosphotransfer (15, 16). In addition, the cysteine directly preceding this His residue in HpeH is serine in CreH and phenylphosphate synthase. We therefore hypothesized that substitution of His581 would abolish phosphotransferase activity, while substitution of Cys580 would attenuate activity. To test these hypotheses, three HpeH variants were generated using site-directed mutagenesis: H581Q, C580S, and C580A. In the spectrophotometric assay, the C580 A and C580S variants turned over HAP at rates similar to the wild-type enzyme, although the specific activity of C580 A was slightly higher, while that of the C580S variant was slightly lower (Table 4). The H581Q variant showed no detectable activity above background. This loss of function is consistent with the catalytic mechanisms proposed for RPH, PEP synthase, and PPDK, where a phosphohistidine intermediate is formed, making this catalytic histidine essential for enzyme function.

**Table 4:** Specific activity of WT and variant HpeH with HAP

HpeH	Specific activity^a^
	U/mg HpeH
WT	0.6 (0.06)
C580S	0.5 (0.03)
C580A	1.0 (0.1)
H581Q	ND

a Activity measured spectrophotometrically with 0.01 μM HpeH and 0.40 μM HpeI with 100 μM HAP. The values represent the mean and standard deviations of three technical replicates. ND, not detected.

## Discussion

This study establishes HpeHI as a two-component phenolic dikinase that catalyzes the efficient ATP-dependent phosphorylation of HAP and AV. The enzyme also catalyzes the phosphorylation of a broad range of analogs, including 4-hydroxybenzaldehyde, cresol, and vanillin (Table 3). Nevertheless, HpeHI has the highest substrate specificities for HAP and AV, two growth substrates of GD02, the strain from which the enzyme was isolated. 4-Hydroxy-benzaldehyde and vanillin are chemically like HAP and AV, both with a formyl substituent instead of the methyl ketone. This -H for -CH_3_ difference results in a >20-fold reduction in enzyme specificity, suggesting there are important structural contacts at this region that could stabilize the preferred substrates in the active site. Although GD02 grows on vanillin, its genome encodes two predicted vanillin dehydrogenases, strongly suggesting that HpeHI is not involved in vanillin catabolism by GD02 (8). Finally, HpeHI has low specific activity for acetosyringone, a related compound on which GD02 does not grow, but which is a growth substrate for *S. lignivorans* SYK-6, a strain that harbours a pathway homologous to the Hpe pathway (10). This suggests that the dikinase plays an important role in determining the specificity of the catabolic pathway.

HpeHI shares several properties, including mechanistic characteristics, with CreHI of *C. glutamicum* and PpsAB of *T. aromatica*, homologs that initiate the catabolism of 4-methylphenol and phenol, respectively. For example, the pH optimum of the phenol phosphorylation reaction catalyzed by PpsAB is approximately 8.5 (12), consistent with the activity of HpeHI increasing from pH 7.5 to 8.5 (Fig. S3). This pH profile might reflect the possibility that these enzymes bind their phenolic substrate in the deprotonated form. More importantly, the data indicate that HpeHI shares the same mechanism as PpsAB (20), in which the PpsB presents ATP to PpsA, facilitating nucleophilic attack of the β-phosphate by His560 (corresponding to His581 in HpeH) and resulting in the pyrophosphorylation of this residue. The γ-phosphate is then hydrolyzed, yielding a β-phosphorylated enzyme intermediate that phosphorylates the phenolic substrate (12, 20). A similar mechanism has been proposed for RPH (15). Interestingly, the sub-micromolar *K*_M_ values of HpeHI for its preferred phenolic substrates agree well with that of RPH for RIF (21) but are a couple of orders of magnitude lower than those of PpsAB (12) and CreHI (11). The SEC-MALS and mass photometry analyses performed under a variety of conditions indicate that HpeH and HpeI do not form a stable complex. However, the inverse dependence of HpeHI activity on ionic strength (Fig. S4) is consistent with these components forming a transient complex that is stabilized by electrostatic interactions, as suggested by the AlphaFold analysis (Fig. S16).

The dependence of HpeHI activity on divalent cations, including the requirement of Mg^2+^ and Mn^2+^ for optimal function (Fig. 2), is consistent with what has been reported for related enzymes. However, for CreHI, PpsAB, and PEP synthase, Mg^2+^ is essential for activity, while in the case of HpeHI, Mn^2+^ alone can partially compensate for Mg^2+^ (11, 12, 22). Moreover, higher concentrations of Mg^2+^ and Mn^2+^ inhibit PEP synthase, and, more generally, other transition metals can support kinase activity (23), similar to how Co^2+^ and Ni^2+^ stimulate HpeHI activity (Table 1). In biological systems, Mn^2+^, Co^2+,^ and Ni^2+^ often have an octahedral coordination geometry, while Zn^2+^ is often tetrahedral (24). The inhibitory effect of Zn^2+^ on the reaction may indicate that the coordination geometry of the metal ion is important for the rate enhancement. Interestingly, the conditions for optimal HpeHI activity resemble the cellular concentrations of Mg^2+^ and Mn^2+^, which are typically present at millimolar and micromolar concentrations, respectively (25, 26). Further studies are required to understand how Mn^2+^ stimulates the catalytic activity of HpeHI.

The structural data indicate that HpeHI is a two-component homolog of RPH, a single component enzyme involved in antibiotic resistance (15, 16). More particularly, the structural data indicate that HpeI corresponds to the ATP-grasp domain of RPH while HpeH harbors the swivel and RIF-binding domains of RPH. Homologous two-component dikinases include CreHI and PpsAB, which initiate the aerobic catabolism of 4-methylphenol and the anaerobic catabolism of phenol, respectively (11, 12). Previous studies have noted homology between PpsAB and the central metabolic enzymes PEP synthase and PPDK. Our analysis indicates that this homology is limited to the ATP-grasp domain and the swivel domain; the second substrate-binding domain of PEP synthase and PPDK has a structural fold not present in HpeHI. Finally, the previously noted homology between PpsAB and EI of the phosphotranferase system is also limited to the swivel domain. Nevertheless, the occurrence of these domains in different contexts highlights how these components were recruited for use in different contexts. Finally, recognition of the two-component subfamily of small-molecule dikinases involved in aromatic compound catabolism should facilitate the identification of homologs involved in other processes.

The amino acid residues surrounding the catalytic histidine of PEP synthase are highly conserved in all species, composed of GGX**T**S/C**H**AAI/VI/VA/SR, with the regulatory threonine and catalytic histidine in bold. Mutation of the histidine is expected to abolish phosphotransferase activity, while mutation of the preceding amino acid (typically cysteine) can affect specific activity and binding of regulatory proteins (19). This conserved motif occurs in the RPH family of enzymes within 50 residues of the C terminus (21). Replacing the catalytic His827 with alanine in RPH from *Bacillus cereus* ATCC 14579 led to a loss of RIF resistance in *E. coli* and eliminated *in vitro* RPH activity. Similarly, the H581Q mutation abolished activity of HpeH, with no enzyme activity detected (Table 4). This conserved histidine is thought to function as both a phosphate acceptor and donor and is necessary for creating a phosphohistidine intermediate. These results suggest that HpeHI is mechanistically related to PEP synthase, RPH, and PpsAB. Notably, both PpsA and CreH contain serine residues directly preceding the catalytic histidine. However, the C580S mutant of HpeH exhibited reduced activity compared to both the WT HpeH and the C580A mutant. The increase in turnover rate exhibited by the C580 A mutant could be due to a reduction in steric hindrance around the active site. These findings highlight the critical role of conserved residues near the catalytic histidine and suggest potential avenues for further research into enzyme regulation and function.

Two-component small-molecule dikinases are involved in the catabolism of a range of phenolic compounds. However, it is unclear why the catabolism of these compounds is initiated by their phosphorylation. The low *K*_M_ values of HpeHI for its HAP and AV suggest that these dikinases may enable scavenging of these substrates from the environment. More specifically, these compounds likely diffuse passively into the cell (27). Their phosphorylation would prevent their diffusion back out and would help maintain a concentration gradient favorable to diffusion into the cell. Further work on this relatively unexplored group of kinases should provide valuable insight into their biological role, catalytic mechanism, and involvement in other cellular processes.

## Experimental procedures

### Chemicals, reagents, and bacterial strains

All reagents were of analytical grade unless otherwise noted. Reagents were obtained from Sigma-Aldrich and Fisher Scientific. Media were prepared using water purified on a Barnstead NANOpure UV apparatus to a resistivity of greater than 16 MΩ/cm.

### DNA manipulation

DNA was manipulated, propagated, and purified using standard techniques (28). Chemically competent *E. coli* DH5α (New England Biolabs) was transformed with plasmid DNA by heat shock at 42 °C for 30 s. Q5 High-Fidelity DNA Polymerase, restriction enzymes, Q5 Site-Directed Mutagenesis kit, and NEBuilder HiFi DNA Assembly Master Mix were purchased from New England Biolabs. Oligonucleotides were purchased from Azenta Life Sciences. Sanger sequencing of constructs was performed by Azenta Life Sciences. The whole plasmid sequencing of key constructs was performed by Plasmidsaurus.

### Construction of hpeH and hpeI expression vectors

The *hpeI* gene was amplified from extracted GD02 genomic DNA using oGD44 and oGD45 (Table S4). This DNA fragment was cloned into pET15b linearized by *Nde*I/*Hind*III to yield pGD106 (Table S5). The *hpeH* gene was amplified from extracted GD02 genomic DNA oGD19 and oGD20. This DNA fragment was cloned into pET28a linearized by *Nde*I/*Xho*I to yield pGD101. To produce variant HpeHs, plasmids pGD108-pGD110 were generated by site-directed mutagenesis using pGD101 as template.

### Protein production and purification

HpeH and HpeI were produced heterologously as N-terminal polyHis-tagged (Ht-) proteins using *E. coli* BL-21 λ (DE3) (Invitrogen) containing plasmids pGD101, pGD106, pGD108, pGD109 or pGD110. Freshly transformed cells were grown at 37 °C shaking at 200 RPM in LB supplemented with 100 mg L^−1^ carbenicillin or 50 mg L^−1^ kanamycin until the culture reached an OD_600_ between 0.4 to 0.6. Expression was induced with 0.5 mM isopropyl β-D-thiogalactopyranoside, and the cells were incubated at 30 °C for an additional 16 h. Cells from 1 L of culture were cooled on ice, pelleted by centrifugation at 4 °C, washed in equilibration buffer containing 50 mM sodium phosphate and 300 mM NaCl, pH 8.0, and frozen at −70 °C until use. Pellets were thawed on ice and suspended in 20 ml of equilibration buffer supplemented with 2 tablets of protease inhibitor (cOmplete Mini), ∼1 mg lysozyme and ∼1 mg DNaseI. Cells were lysed at 4 °C using an EmulsiFlex-C5 homogenizer (Avestin) and cellular debris was pelleted by centrifugation at ∼26,000 RCF. The soluble fraction was filtered (0.45 μm) and proteins were purified using immobilized metal affinity chromatography (Ni-NTA, Thermo Fisher Scientific, Waltham, MA, USA). The protein-loaded resin was first washed with an equilibration buffer, followed by a wash with equilibration buffer containing 25 mM imidazole, and eluted with 15 ml of equilibration buffer with 250 mM imidazole. Fractions containing Ht-HpeH or Ht-HpeI, as determined by sodium dodecyl sulfate polyacrylamide gel electrophoresis (SDS-PAGE), were pooled, and the buffer was exchanged using NMWCO Amicon Ultra Centrifugal Filter Unit (10 kDa for HpeI, 30 kDa for HpeH) with buffer containing 20 mM Tris pH 8.0, 10% glycerol and 0.3 mM tris(2-carboxyethyl)phosphine (TCEP) for histidine tag cleavage by tobacco etch virus protease [TEV]. Cleavage was performed in reactions containing 20 mM Tris pH 8.0, 10% glycerol, 0.3 mM TCEP with a molar ratio of 50:1 (HpeH:TEV) or 20:1 (HpeI:TEV). Reactions were incubated at room temperature for 6 to 8 h and then at 4 °C overnight. The cleaved preparation was run over a Ni-NTA column with 2 ml resin and the flow-through was collected and run on SDS-PAGE to verify complete cleavage. The cleaved protein was concentrated with a NMWCO Amicon Ultra Centrifugal Filter Unit (10 kDa for HpeI, 30 kDa for HpeH), flash frozen in liquid N_2_ and stored at −70 °C. Protein concentration was determined by Micro BCA assay (Thermo Fisher Scientific) with bovine serum albumin as a standard.

### Determination of extinction coefficients

The extinction coefficients of HAP and PAP were determined at 30 °C at pH 7.5, 8.0 and 8.5. The compounds were dissolved at a concentration of 100 μM in buffer containing 2.0 mM DTT, 2.0 mM MgCl_2_, 1.0 mM MnCl_2_ and 20 mM HEPPS (for pH 8.0 and 8.5) or 20 mM HEPES (for pH 7.5). Spectra were recorded using a Cary 5K UV-Vis-NIR spectrophotometer (Agilent Technologies) outfitted with a thermostatted cuvette holder. For reactions involving HAP, the Δε_325_ was calculated by subtracting the ε_325_ of PAP from the ε_325_ of HAP. For reactions involving AV, the Δε_314_ and Δε_341_ values were determined similarly at pH 7.5 and pH 8.0, respectively using the corresponding extinction coefficients of AV and PAV. Extinction coefficients for the reactions containing vanillin (Δε_340_ = 12.4 mM^−1^ cm^−1^) and 4-hydroxybenzaldehyde (Δε_330_ = 11.6 mM^−1^ cm^−1^) were determined by recording the difference spectra before and after complete reaction with 2 μM HpeH and 2 μM HpeI. Reactions were quenched with 10% vol/vol acetic acid, and samples were analyzed by LC-MS to verify that < 1% of substrate remained.

### HpeHI activity assay

Assays were performed in a total volume of 3.0 ml containing 100 μM HAP, 500 μM ATP, 2.0 mM MgCl_2_, 1.0 mM MnCl_2_, 0.40 μM HpeH and 0.80 μM HpeI at 30 °C. In evaluating the effect of pH on reaction rate, buffers were 20 mM HEPPS (at pH 8.0 and 8.5) or 20 mM HEPES (pH 7.5) and contained 2.0 mM DTT. In evaluating the effect of ionic strength, the reaction buffer contained 10 mM NaOH, 0.50 mM TCEP and HEPES added to pH 7.5. Ionic strength was varied by adding NaCl to between 0 and 490 mM. Significant differences were determined using an unpaired two-tailed *t* test. In evaluating the effect of divalent cations, the reaction buffer contained 10 mM NaOH, 0.50 mM TCEP, and the pH was adjusted to 7.5 using HEPES. The ionic strength was maintained at 29 mM using NaCl. Alternative metal ions were assessed by replacing 50 μM MnCl_2_ with equal concentrations of ZnCl_2_, CoCl_2_, and NiCl_2_.

### Calculation of specific activity

Reactions were performed in triplicate and initiated by addition of aromatic substrates. Reaction progress curves were monitored for ≥4 min at the λ_max_ of HAP or AV using a Cary5000 spectrophotometer with a thermostatted cuvette. Initial rates were calculated using the change in absorbance at 325 nm plotted against time over 30 s. A background rate was measured by performing the reaction in the absence of HpeH. This background rate was subtracted from rates measured in the presence of HpeH or its variants.

### Steady-state kinetics

The standard assay for HpeHI activity contained 0.05 μM HpeH, 0.10 μM HpeI, and 500 μM ATP. The buffer contained 10 mM NaOH, 0.50 mM TCEP, 3.9 mM NaCl, 2.0 mM MgCl_2_ and 0.025 mM MnCl_2_ (*I =* 20 mM) with HEPES added to pH 7.5 at 30 °C. Plots were generated using GraphPad Prism and steady-state kinetic parameters were determined by fitting the Michaelis-Menten equation to the data by least-squares fitting using LEONORA analysis software (29).

### HpeHI activity assays with additional aromatic substrates

Reactions were performed using the buffer described for the standard assay with 100 μM of each substrate in 1.0 ml total volume containing 0.20 μM HpeH and 0.40 μM HpeI. Reactions were run for 1 to 30 min at 30 °C, depending on the substrate, and quenched with 10% vol/vol acetic acid. Rates were calculated by quantifying either production of the phosphorylated product for HAP, AV, and phenol, or substrate depletion for other substrates, using authentic standards. LC-MS analyses were performed using an Agilent 1290 Infinity II UHPLC in line with an Agilent 6546 Q-TOF with a dual AJS ESI source. For all compounds excluding cresol, 2.0-μL samples were injected onto a Zorbax Eclipse Plus c18 RRHD column (50 mm × 2.1 mm, 1.8 μM) and run on a 12-min linear gradient from 5 to 100% methanol, with 0.10% formic acid at 0.25 ml min^−1^. Cresol was monitored by injecting 5.0 μl onto a Zorbax Extend-C18 RRHT column (50 mm × 2.1 mm, 1.8 μm) and run on a 17-min linear gradient from 5 to 90% methanol with 10 mM ammonium acetate pH 9.0. MS parameters in negative ionization mode were as follows: capillary voltage, 3500 V; nozzle voltage, 1000 V; drying gas temp, 250 °C; drying gas flow rate, 10 L min^−1^; sheath gas temperature, 300 °C; sheath gas flow rate 12 L min^−1^, nebulizer pressure, 40 psi; fragmentor voltage, 100 V. Parameters for positive ionization mode were the same, except the nozzle voltage, 500 V. MS/MS spectra were collected for selected ions with 10-, 20-, and 40-V collision energies. Data were collected using MassHunter Workstation Version 10 and processed using Profinder Version 10.0.2.

### Phosphate exchange by HpeH

Reactions were performed using the standard assay buffer at 25 °C. The reaction contained 50 μM PAP, 50 μM AV, and 1.0 μM HpeH in 400 μl volume. Supplementation with 1.0 μM HpeI and/or 0.5 mM AMP-PNP were also tested. Reactions were quenched with 10% vol/vol acetic acid at 15 and 70 min and centrifuged for 10 min at max speed in a microcentrifuge. PAV was quantified by LC-MS with a PAV standard curve, as described above.

### Crystallography

HpeI was further purified after immobilized metal affinity chromatography by size-exclusion chromatography using a HiLoad 16/600 Superdex 200 pg column equilibrated in 20 mM Tris pH 7.5, 150 mM NaCl, 3.0 mM β-mercaptoethanol, and concentrated to 10 mg mL^−1^. Crystallisation conditions were identified using the Mosquito crystal liquid handling robot (SPT Labtech) in SWISSCI 3-lens low profile plates, using various sparse matrix screens. HpeI crystals grew in condition C4 (25% PEG 1500 and 0.1 M PCTP pH 7.0) of the PACT screen (Molecular Dimensions). Crystals were cryoprotected in crystallization buffer containing 25% glycerol and flash frozen in liquid nitrogen. Diffraction data were collected at beamline I04 at the Diamond Light Source (Didcot, UK). The data were anisotropic and were processed using STARANISO (30). The structure was solved by molecular replacement with MolRep (31), using an AlphaFold model (32, 33). Model building was performed in Coot (34) and the structure was refined with Refmac5 (35). MolProbity (36) was used to evaluate the final model and PyMOL (Schrödinger, LLC) was used for protein model visualization. Searches for structural protein homologs were performed with the DALI server (37). RMSD values were calculated using the combinatorial extension [CE] alignment plugin in PyMOL (38).

### Analysis of protein oligomeric state

The oligomeric state and potential HpeHI complex were examined using size-exclusion chromatography coupled to a multi-angling light scattering detector (SEC-MALS) and mass photometry. SEC-MALS was performed using an Agilent 1260 HPLC equipped with a UV detector and in-line with a Superdex 200 30/100 column operated at a flow rate of 0.2 ml min^−1^. 50 microliters of HpeH (12.5 μM), HpeI (25 μM) or a combination of the two were incubated at room temperature for at least 20 min and injected into the column. The mobile phase was consistent with the standard assay buffer described above, containing 10 mM Hepes pH 7.5, 0.50 mM TCEP, 20 mM NaCl, 2.0 mM MgCl_2_, 0.25 mM MnCl_2_, but to mitigate interaction with the column resin, the buffer was supplemented with 20 mM NaCl or 200 mM NaCl. The system was attached to a miniDAWN Treos light scattering detector and an Optilab T-rEX differential refractometer detector (Wyatt Technology). Data were collected and analyzed using Astra 6 software.

Mass photometry was performed using a Refeyn TwoMP (Refeyn Ltd) and analyzed with DiscoverMP 3.2. β-Amylase, thyroglobulin, and bovine serum albumin were used for molecular weight calibration. Samples were prepared at a concentration of 20 nM in the standard assay buffer, and 4.0 μl were pipetted on pretreated 6-well sample cassettes applied on a sample carrier slide (Refeyn MP-CON-21008). Droplet dilution was used for auto-focusing. The acquisition time was 60 s.

## Data availability

X-ray data of HpeI are deposited in the Protein Data Base under code 9GOJ.

## Supporting information

This contains supporting information.

## Conflict of interest

The authors declare that they have no conflicts of interest with the contents of this article.
