# Supplementary material for: Characterization of a two-component kinase that initiates the bacterial catabolism of hydroxyphenylethanones
Source: J Biol Chem. 2025 May 8;301(6):110210. doi: 10.1016/j.jbc.2025.110210 (PMC12167813; doi:10.1016/j.jbc.2025.110210)
Supplement: Supporting information [file mmc1.docx]

**SUPPLEMENTARY INFORMATION**

**Experimental procedures** *Determination of extinction coefficients* Spectra of HAP and PAP were recorded in 20 mM HEPES (pH 7.5) or HEPPS (pH 8.0 and 8.5) containing 2 mM DTT, 2 mM MgCl_2_, 1 mM MnCl_2_. Under these conditions, the spectrum of PAP was relatively unaffected by pH, with λ_max_ of 269 nm and ε_269_ of 11 mM^-1^ cm^-1^ (**Figure S2**). By contrast, the spectrum of HAP was strongly pH-dependent, displaying peaks at 275 and 325 nm that presumably reflect the phenol and phenolate forms of HAP, respectively. Although the relative intensity of the peak at 325 nM increased with pH, at the three tested pH values, the maximum difference in absorption between HAP and PAP was at 325 nm. Therefore, HpeHI activity was monitored at this wavelength. The extinction coefficient, ε, of HAP and PAP were determined at pH 7.5, 8.0 and 8.5 and used to calculate the Δε_325_ for each pH (**Table S1**). These values were used to calculate the activity of HpeHI for HAP in a continuous assay. Analogously, the Δε_314_ for AV and PAV was 2.85 mM^-1^ cm^-1^ at pH 7.5. Since the phosphorylated products of 4-hydroxybenzaldehyde and vanillin were not readily available, UV-Vis spectra were recorded at pH 7.5 before and after treatment with excess HpeHI (2 μM). LC-MS was used to verify that the expected phosphorylated product was produced and that substrates were completely (>99%) depleted at reaction end points. The calculated extinction coefficients for 4-hydoxybenzaldehyde and vanillin phosphorylation were Δε_330_ = 11.6 mM^-1^ cm^-1^ and Δε_340_ = 12.4 mM^-1^ cm^-1^, respectively.


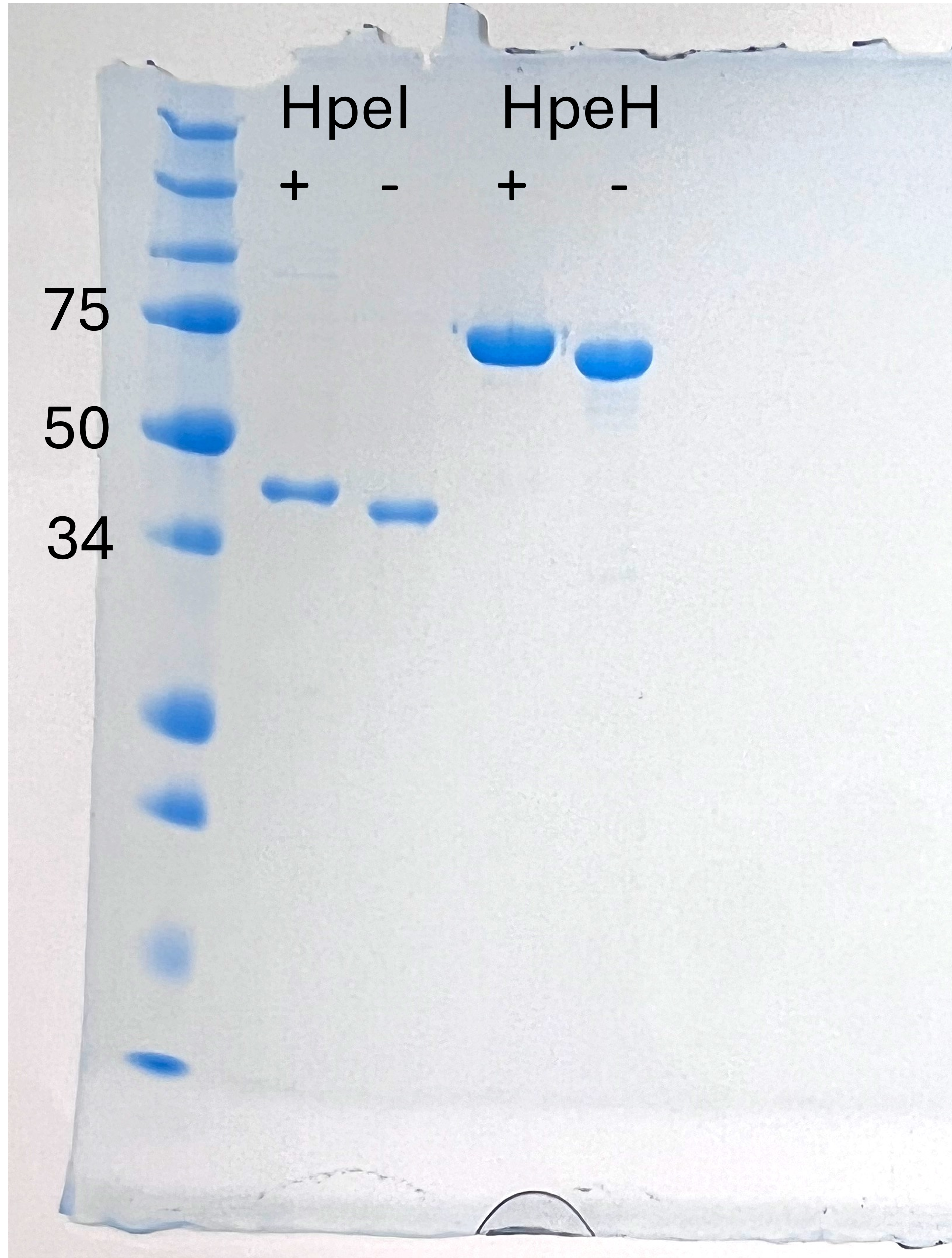
**Figures**

**Figure S1: SDS-PAGE analysis of HpeH and HpeI**. HpeI and HpeH lanes were loaded with ~1 µg of protein either before (+) or after (-) cleavage with TEV protease. Left lane loaded with molecular weight marker (MW of three standards indicated). Calculated molecular weights of HpeH and HpeI are 71.1 and 40.6 kDa, respectively.

**Figure S2: UV-visible spectra of HAP and PAP at different pH values.** Absorption spectra of HAP (blue traces) and PAP (green traces) at pH 7.5, 8.0 and 8.5 at 30 °C. Buffer contained 20 mM HEPPS (pH 8.0 and 8.5) or HEPES (pH 7.5), 2.0 mM DTT, 2.0 mM MgCl_2_, 1.0 mM MnCl_2_. Extinction coefficient calculated from amount of HAP or PAP in sample.

**
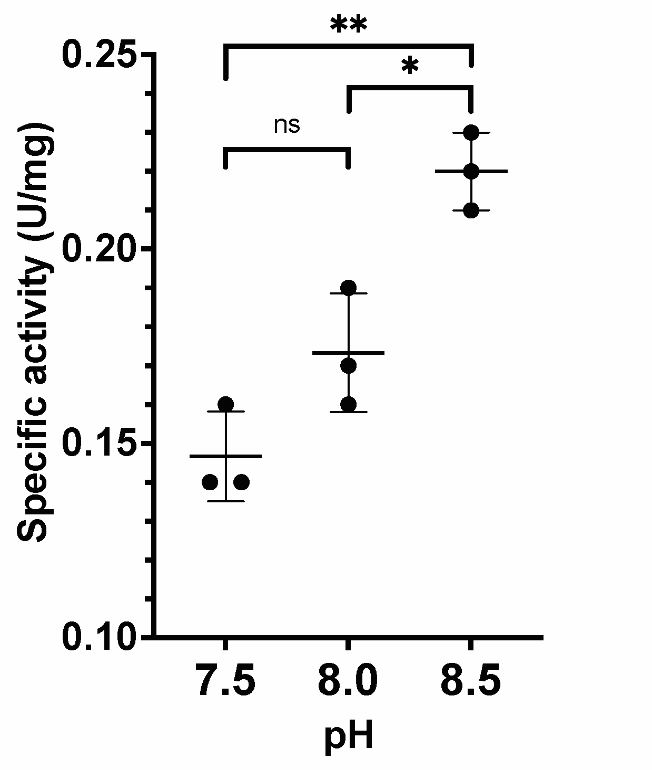
**

**Figure S3: pH-dependence of the rate of turnover of HAP.** Rates of turnover of HAP by HpeHI at pH 7.5, 8.0 and 8.5 at 30 °C with 100 µM HAP, 500 µM ATP, 0.40 µM HpeH and 0.80 µM HpeI. Black circles represent individual replicates. Statistical significance was evaluated using an unpaired t-test: ns, *p* = 0.073; *, *p* $=$ 0.01; **, *p* $=$ 0.001. Error bars represent the standard deviation.


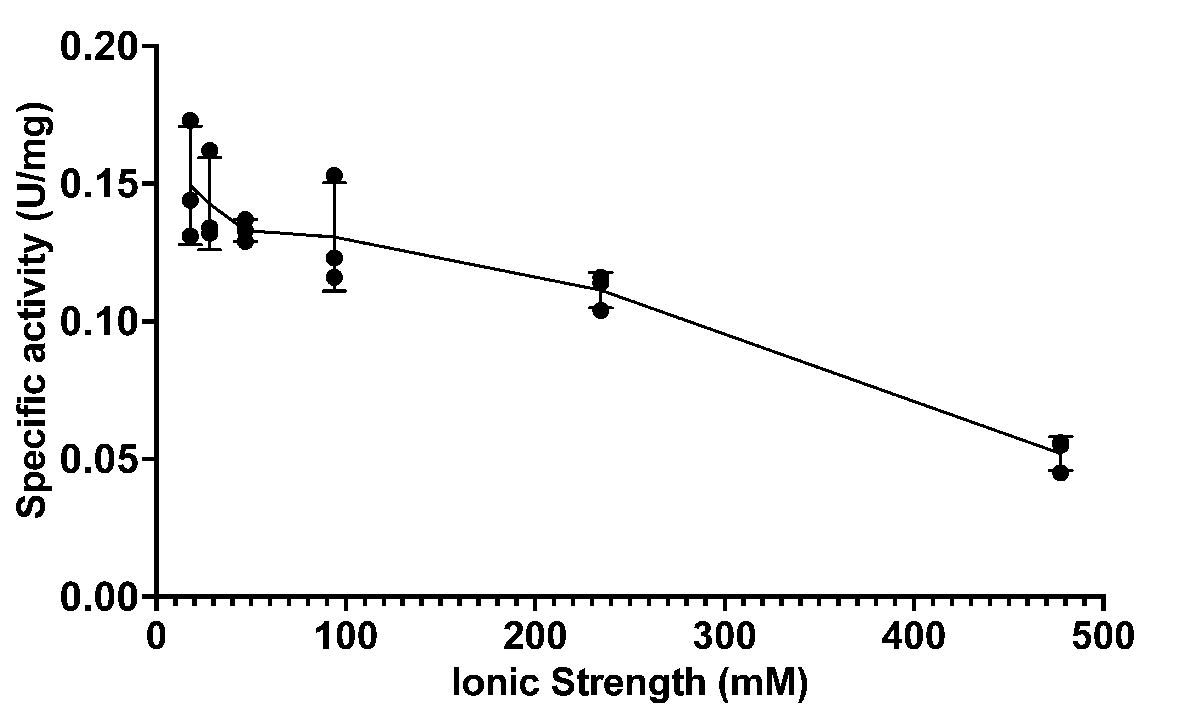


**Figure S4: Dependence of HpeHI activity on ionic strength.** Reactions contained 0.40 µM HpeH, 0.80 µM HpeI, 100 µM HAP and 500 µM ATP, and were performed at 30 °C. The assay buffer contained 10 mM NaOH, 2.0 mM MgCl_2_, 1 mM MnCl_2_, 0.50 mM TCEP. The pH was adjusted to 7.5 with HEPES and the ionic strength was adjusted to the indicated value with NaCl. Reactions were performed in triplicate. Error bars represent standard deviation.


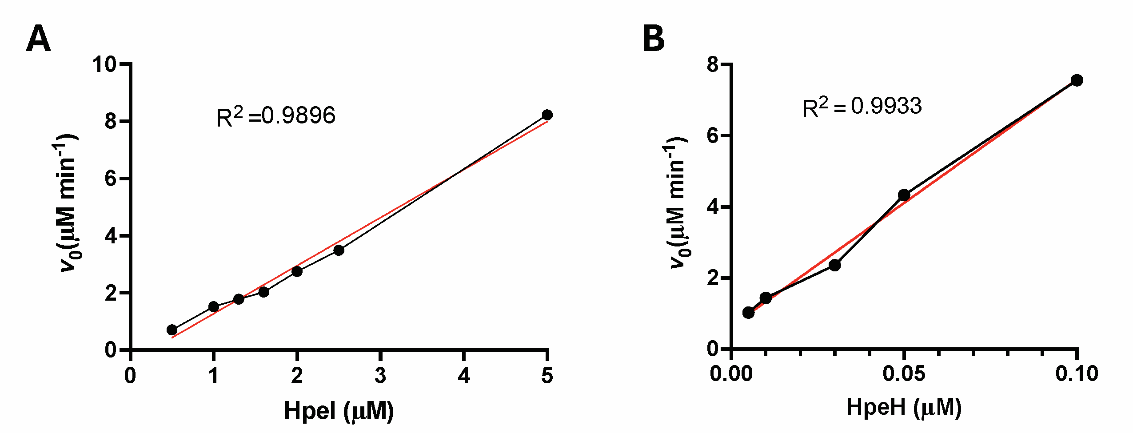


**Figure S5: Dependence of HpeHI activity on enzyme component stoichiometry.** Reactions contained 20 µM HAP, 500 µM ATP and were performed at 30 °C in buffer (*I* = 20 mM) containing 10 mM NaOH, 3.9 mM NaCl, 0.50 mM TCEP, 2.0 mM MgCl_2_, 25 μM MnCl_2_ and HEPES to adjust to pH 7.5. Red lines represent least-squares fit to the data. (**A**) Reactions contained 0.010 µM HpeH. (**B**) Reactions contained 1.0 µM HpeI.


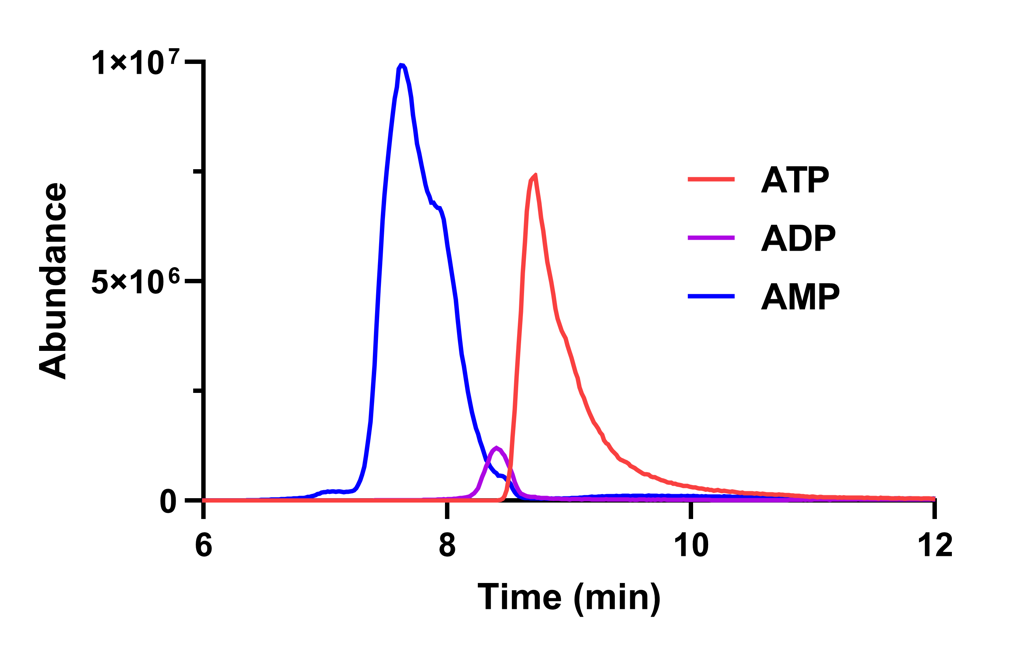


**Figure S6. LC-QTOF analysis of AMP, ADP and ATP levels produced by HpeHI phosphorylation of 4-hydroxyacetophenone.** Traces are extracted ion chromatograms for the target compounds formed after quenching reaction mixtures of HpeHI, 4-hydroxyacetophenone and ATP performed in our standard reaction conditions for determining specific activity.

**
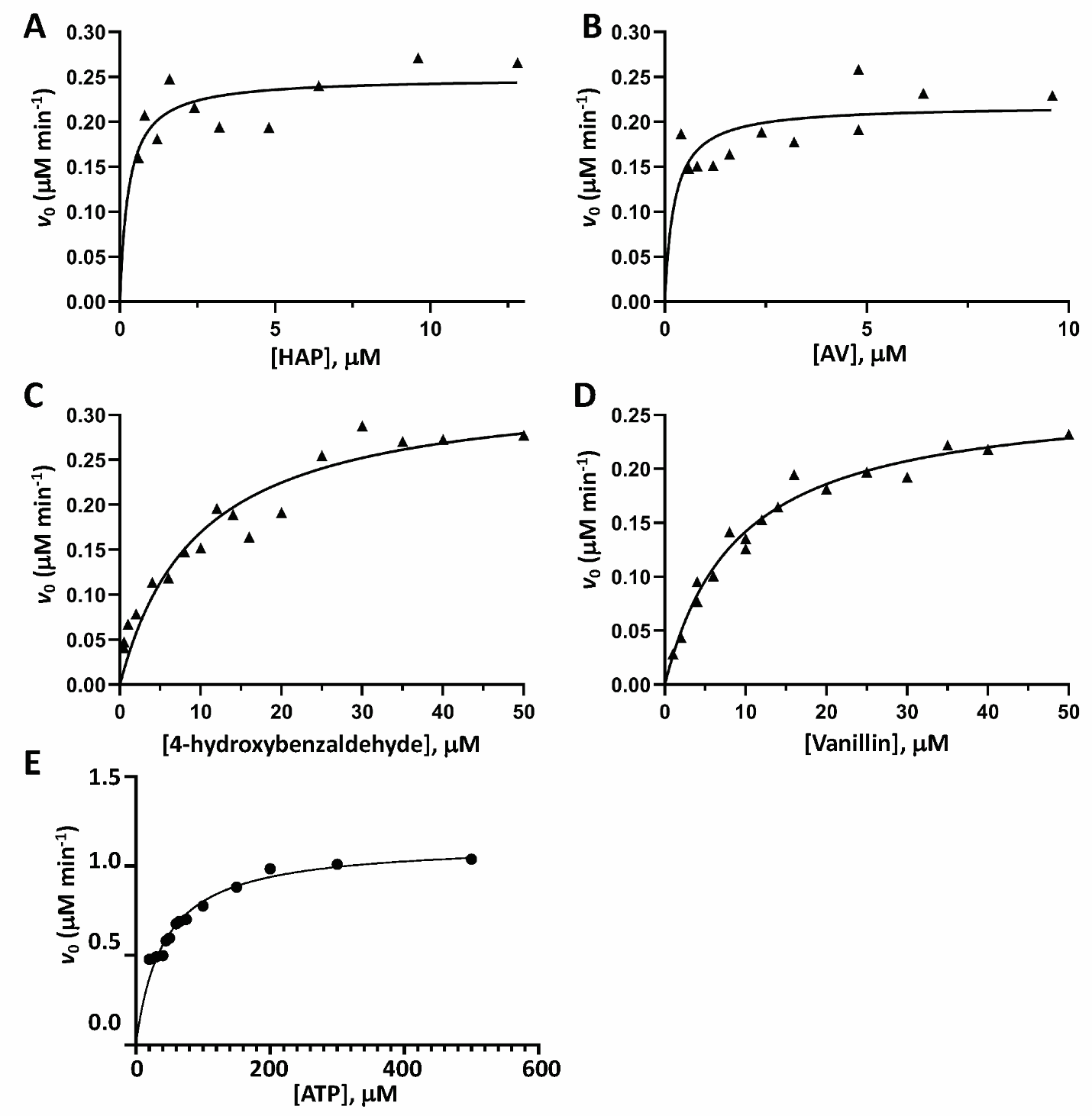
**

**Figure S7: Steady-state kinetic analyses of HpeHI.** Parameters were evaluated for HAP (**A**), AV (**B**), 4-hydroxybenzaldehyde (**C**), vanillin (**D**), and ATP (**E**). Reactions contained 0.05 µM HpeH and 0.10 µM HpeI. Reactions with HAP and AV contained 500 µM ATP. 100 µM HAP was used in reactions determining ATP kinetics. Curves represent a least-squares fit of the Michaelis-Menten equation to the data.


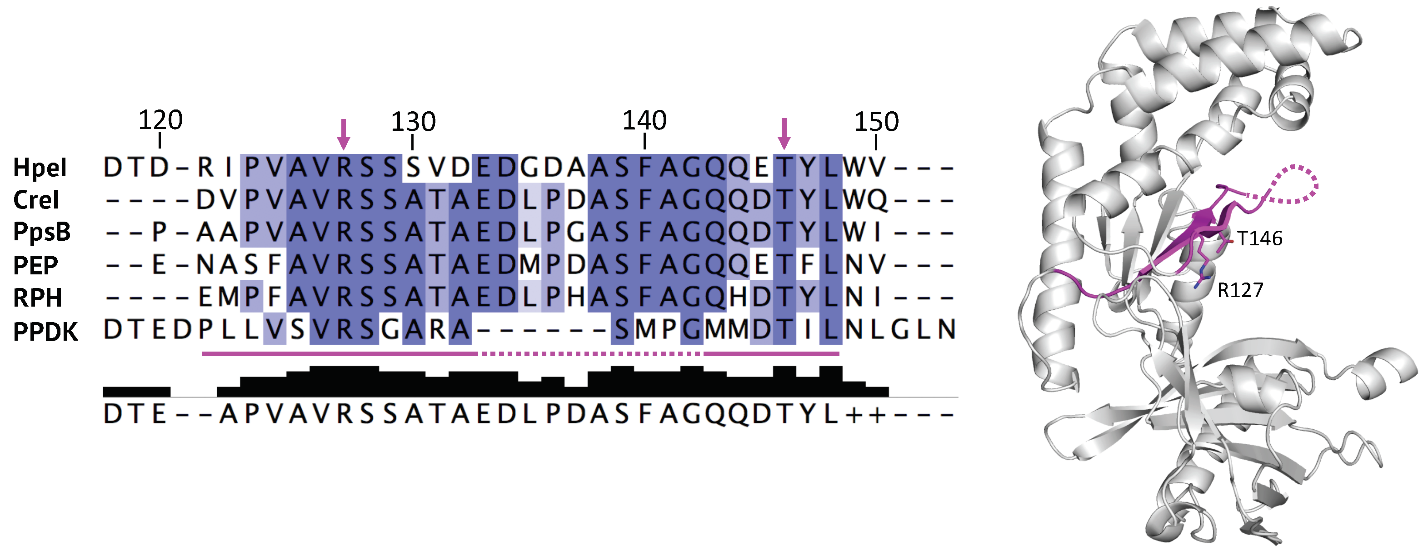


**Figure S8. An ATP binding sequence motif for related ATP grasp domains.** A structure guided sequence alignment was generated using structures of PEP synthase (2OLS), RPH (5HV1) and PPDK (1DIK) with T-coffee Expresso. Sequences are colored by percent identity. The location of the motif is indicated by magenta bar in the alignments and is colored on the HpeI structure in magenta. Two conserved residues that bind the ATP α-phosphate, R127 and T146, are highlighted by magenta arrows and drawn as sidechain sticks on the structure. The dashed line indicates the portion of the HpeI structure that was unmodeled. Proteins included: RPH from *Listeria monocytogenes* strain F2365 (Uniprot ID: A0A0X1KHF9), CreI from *C. glutamicum* (Uniprot ID: Q8NSW3), PpsB from *T. aromatica* (Uniprot ID: A0A2R4BQP6), PEP synthase from *E. coli* (Uniprot ID: P23538), and PPDK from *Clostridium* *symbiosum* (Uniprot ID: P22983).


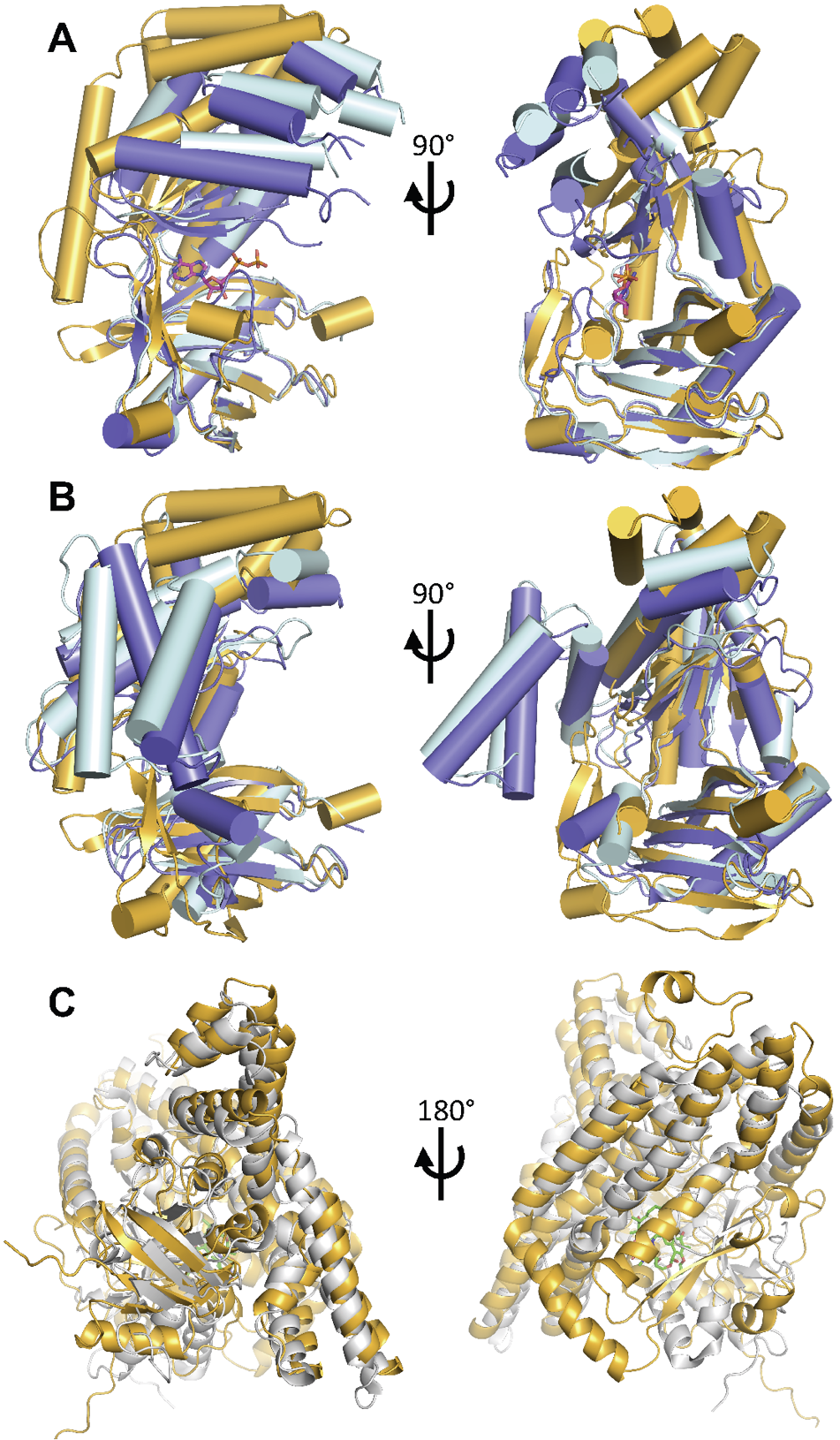


**Figure S9. Superpositions of HpeI and HpeH on structures of homologs**. (**A**) HpeI (orange) is in a more open conformation than apo RPH (2FBT, pale cyan) and the ADP-bound RPH grasp domain (2FBS, dark blue; ADP shown as magenta sticks). (**B**) HpeI (orange) overlayed on the PPDK ATP-grasp domains from *Clostridium* *symbiosum* (2R82, pale cyan) and maize (1VBG, dark blue) (**C**) The AlphaFold HpeH model (orange) on the RPH binding and swivel domains (grey). Rifampin is shown in the RPH substrate binding site as green sticks.


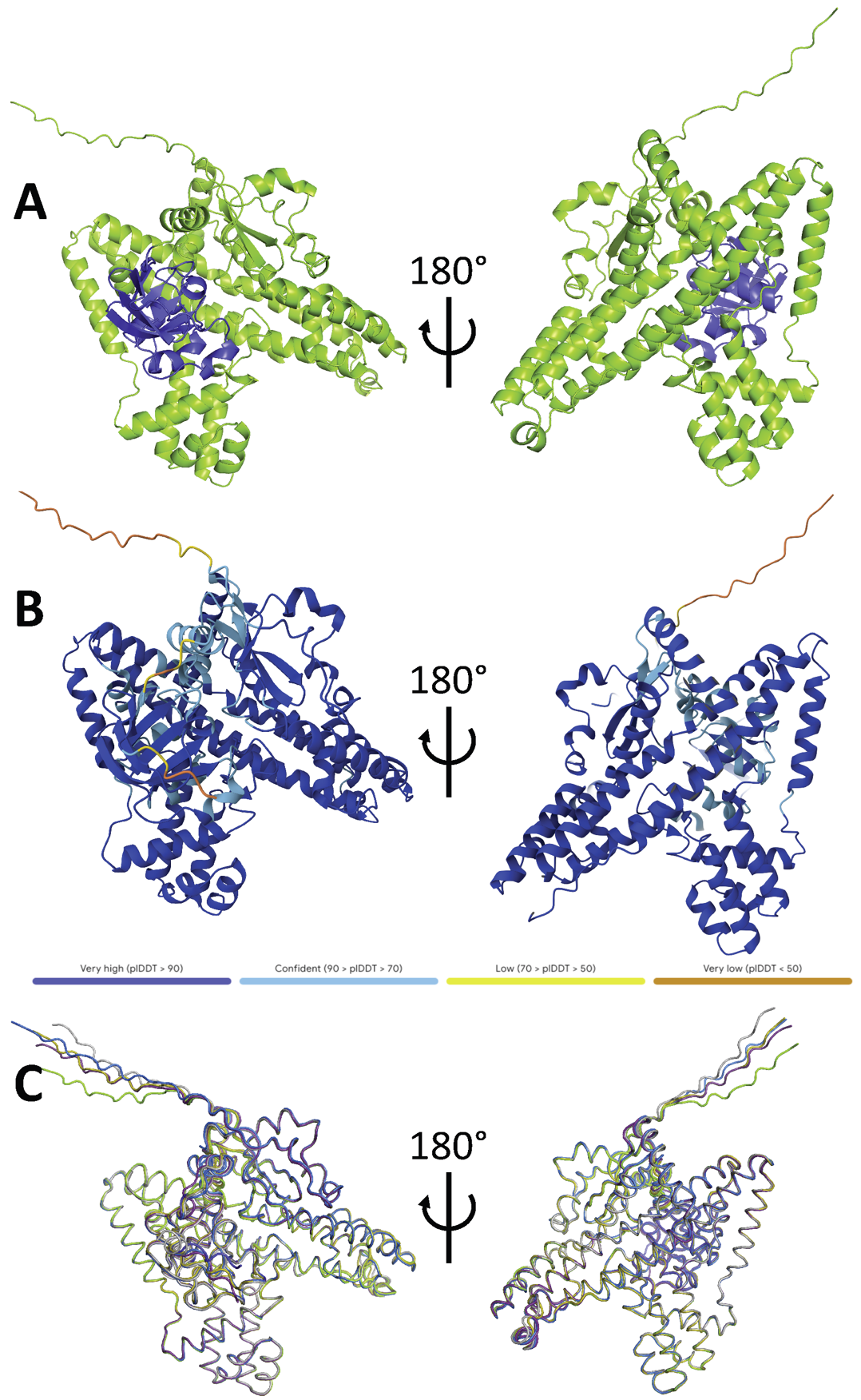


**Figure S10. AlphaFold model of HpeH.** HpeH is shown as a cartoon and colored according to (A) Substrate binding and Swivel domain in green and blue, respectively or (B) by pIDDT score. (C) Overlayed ribbon representations of the top 5 AlphaFold models.


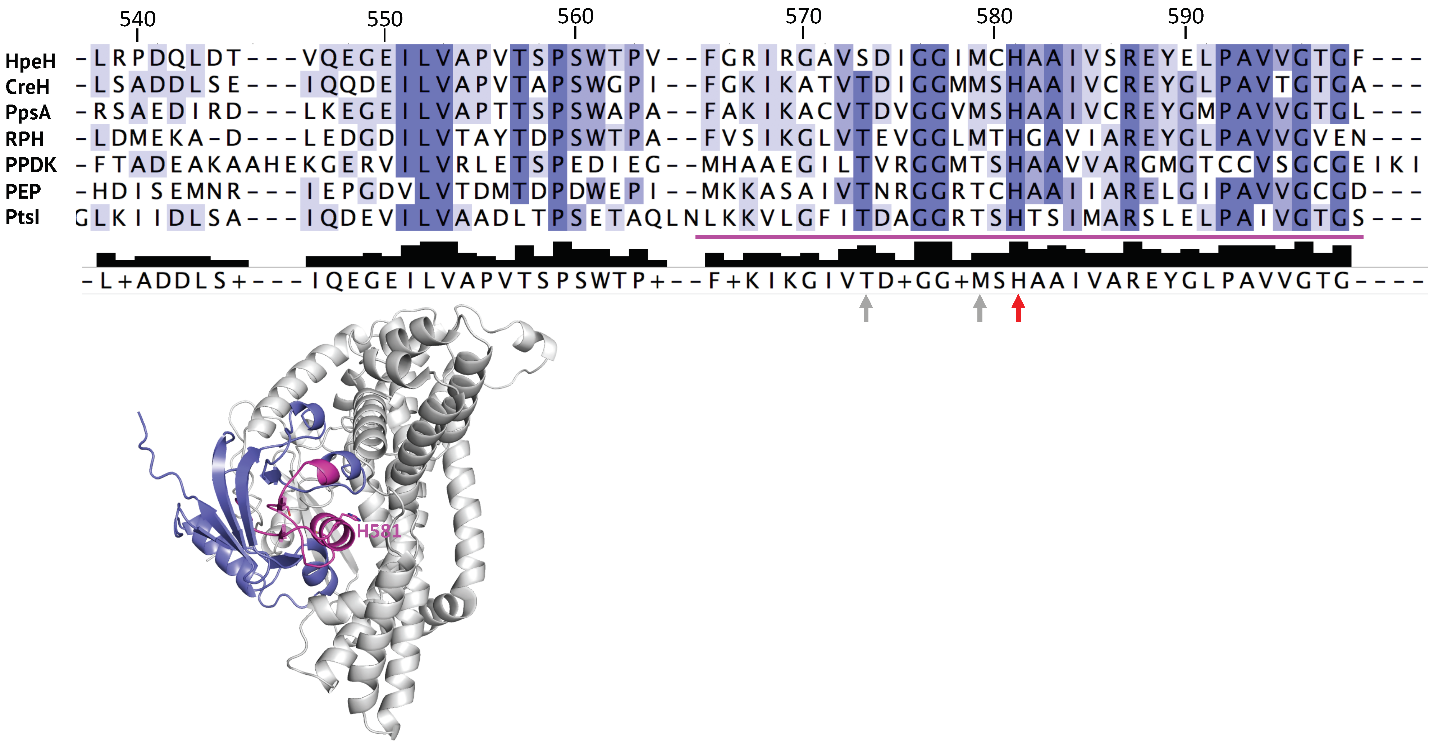


**Figure S11. Structure-guided sequence alignment of HpeH homologs**. Representative sequences from the characterized phenolic substrate kinases, RPH (5HV1), PEP synthase (2OLS), PPDK (1DIK) and PtsI (2HWG) were aligned using T-Coffee Expresso using deposited structures. The sequence alignment shows a conserved region of the Swivel domain (blue in structure) with a key structural motif containing the phosphorylation site, His581 (red arrow), underlined in magenta in the alignment and colored magenta on the structure. The structure used for illustration is the AlphaFold model of HpeH (see Figure S10). Proteins included: RPH from *L. monocytogenes* strain F2365 (Uniprot ID: A0A0X1KHF9, CreH from *C. glutamicum* (Uniprot ID: Q8NSW4), PpsA from *T. aromatica* (Uniprot ID: A0A2R4BQP1, PPDK from *C.* *symbiosum* (Uniprot ID: P22983), PEP synthase from *E. coli* (Uniprot ID: P23538), and PstI from *E. coli* (Uniprot ID: P08839).

**
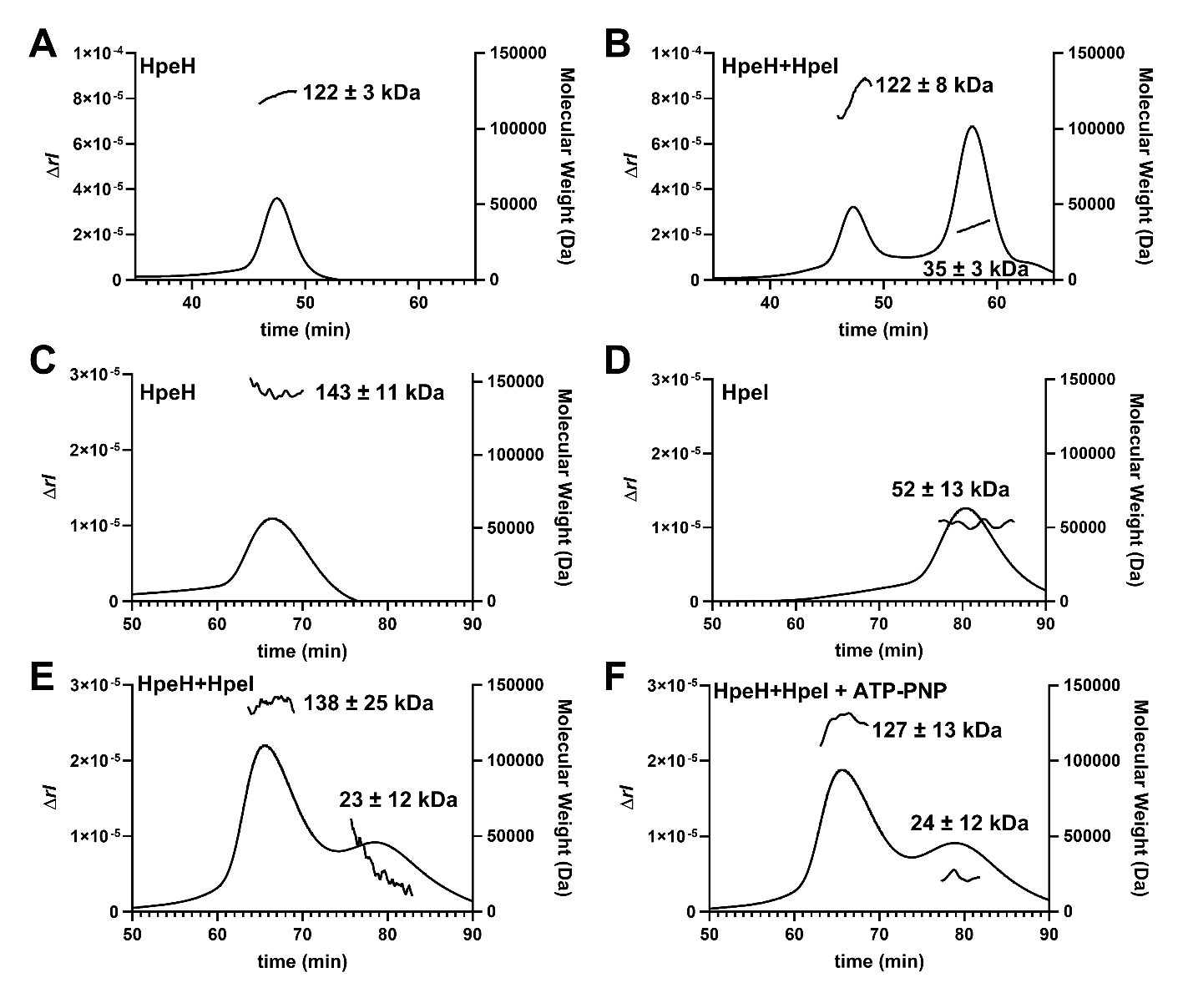
**

**S12. SEC-MALS analysis of HpeH and HpeI.** (**A**) HpeH alone and (**B**) HpeHI in the same buffer used for kinetic characterization, supplemented with 200 mM NaCl. Mixtures contained 20 μM HpeH and 60 μM HpeI. The flow rate was 0.25 ml/min. (**C**) HpeH, (**D**) HpeI (**E**) HpeHI, and (**F**) HpeHI supplemented with 1.0 mM AMP-PNP in the same buffer, instead supplemented with 20 mM NaCl. The lower salt mixtures contained either 12.5 μM HpeH, 25 μM HpeI or both components and were run using a flow rate of 0.20 mL min^-1^. All separations were performed on a Superdex 200 30/100 column. The theoretical mass of HpeH and HpeI are 71.1 and 40.6 kDa, respectively. It is unclear whether HpeH forms a transient dimer in solution or if the flexibility between the binding and swivel domains results in a larger apparent mass.

**
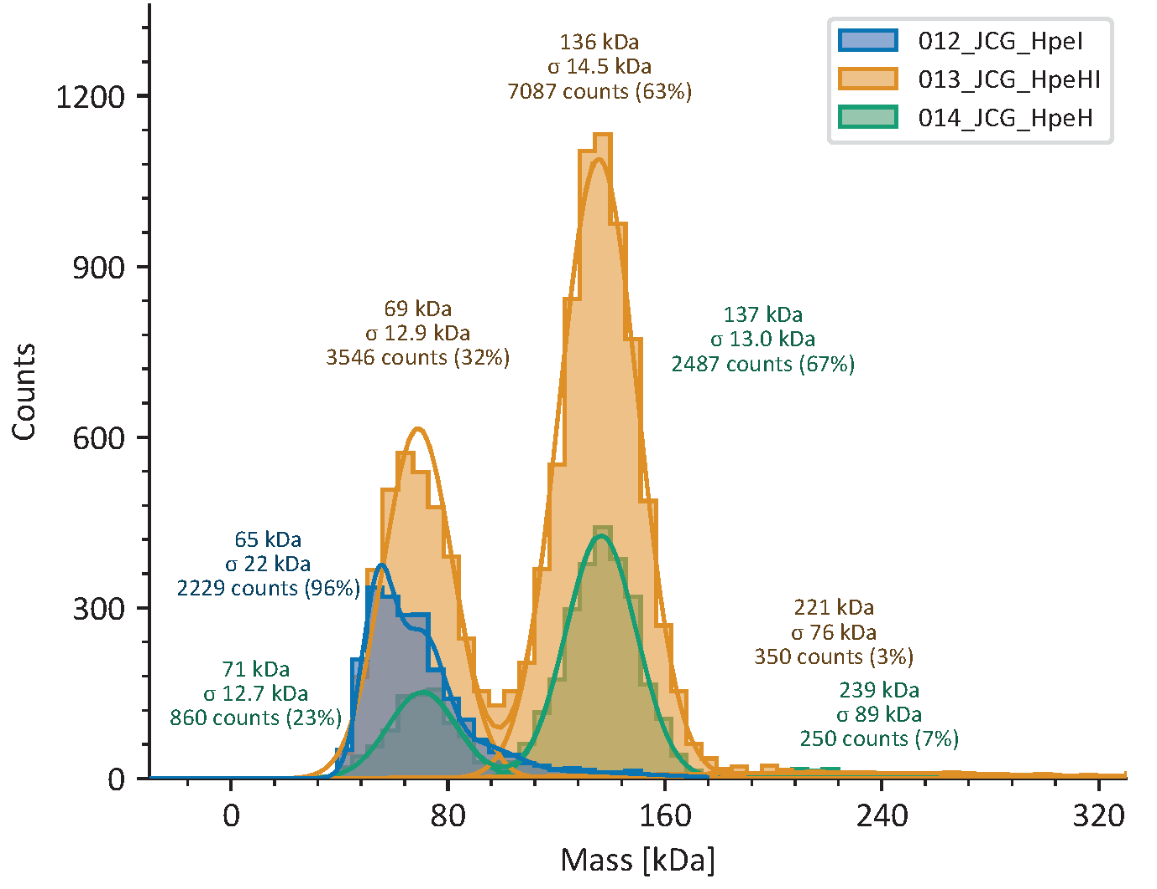
**

**Figure S13. Mass Photometry analysis of HpeH and HpeI oligomeric state.** Measurements were recorded using 20 nM of each protein. The stepped histograms represent the recorded data, and the continuous curves represent the fit to the data. The theoretical mass of HpeH and HpeI are 71.1 and 40.6 kDa, respectively.

**
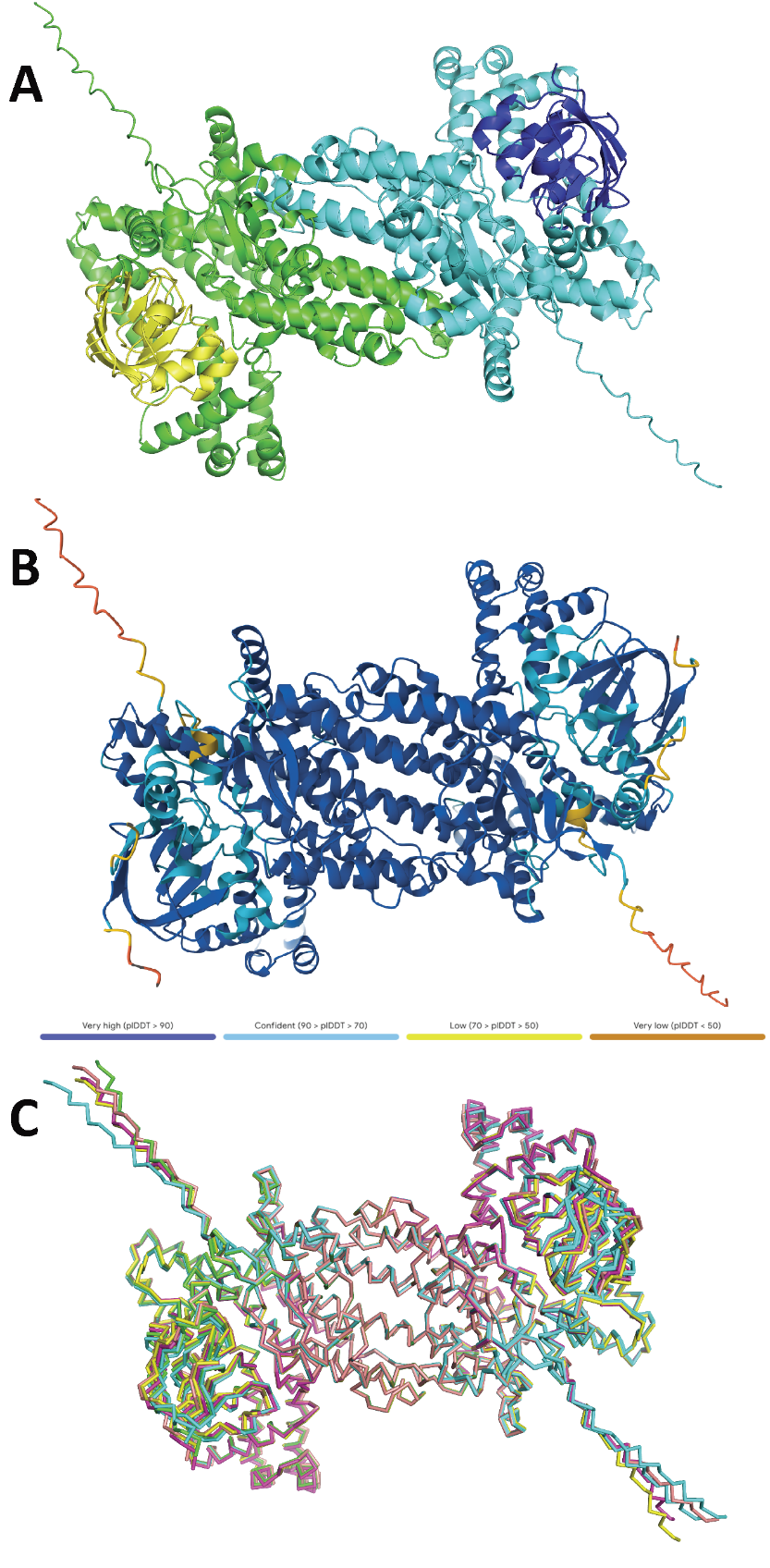
**

**Figure S14. AlphaFold models of a potential HpeH homodimer.** (**A**) The predicted HpeH dimer shown as cartoons with one molecule in green (Substrate binding domain) and yellow (Swivel domain) and the other in cyan (Substrate binding domain) and blue (Swivel domain). (**B**) The predicted homodimer colored according to pIDDT score. (**C**) Overlay of the top 5 AlphaFold models for the dimer shown as ribbons with each dimer colored uniquely.

**
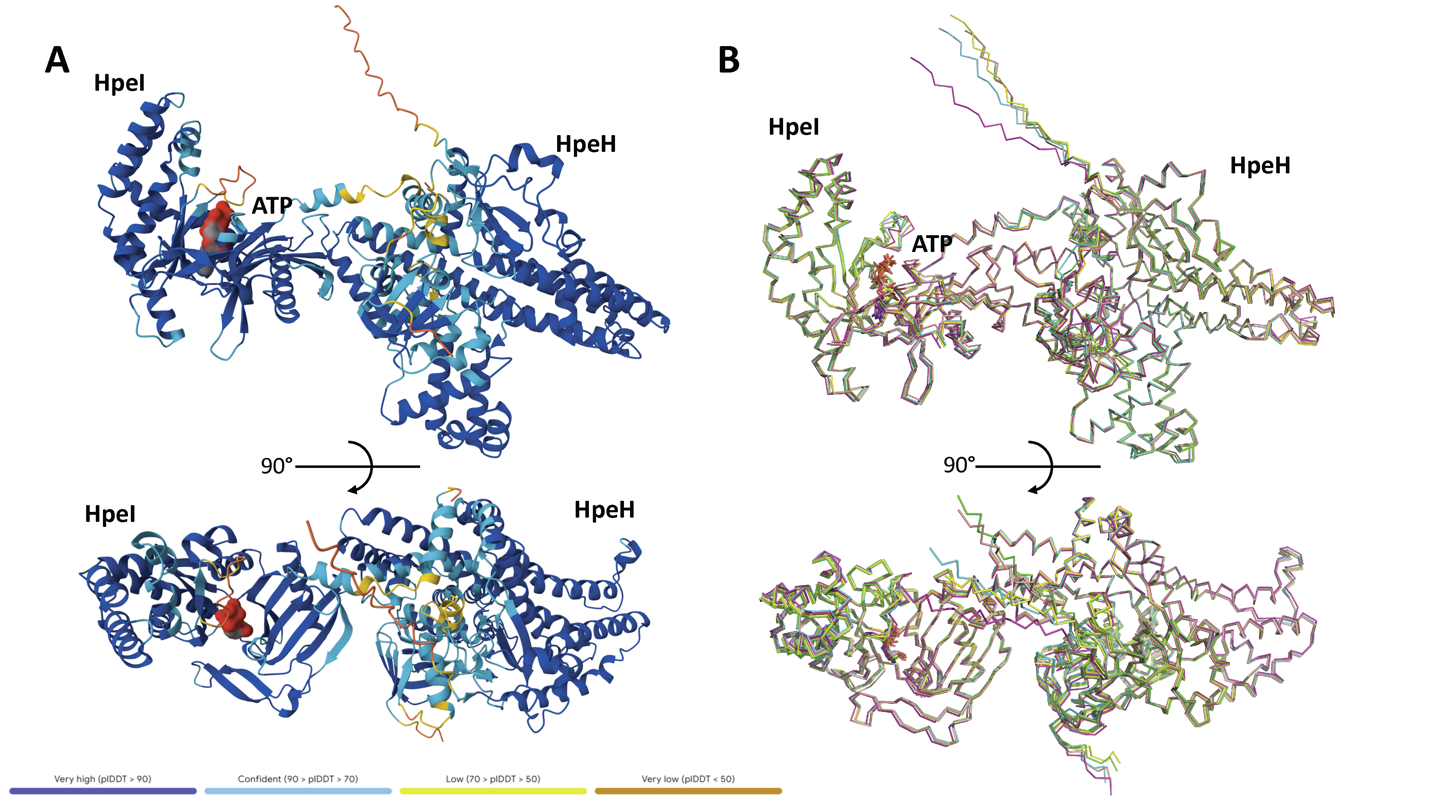
**

**Figure S15. AlphaFold model of the HpeH:HpeI:ATP ternary complex.** Models were generated using AlphaFold 3.0 with one copy of each component. (**A**) pIDDT scored colored onto the ternary complex cartoon. (**B**) Superposition of the top 5 AlphaFold models, shown as ribbons and colored according to model number.

**
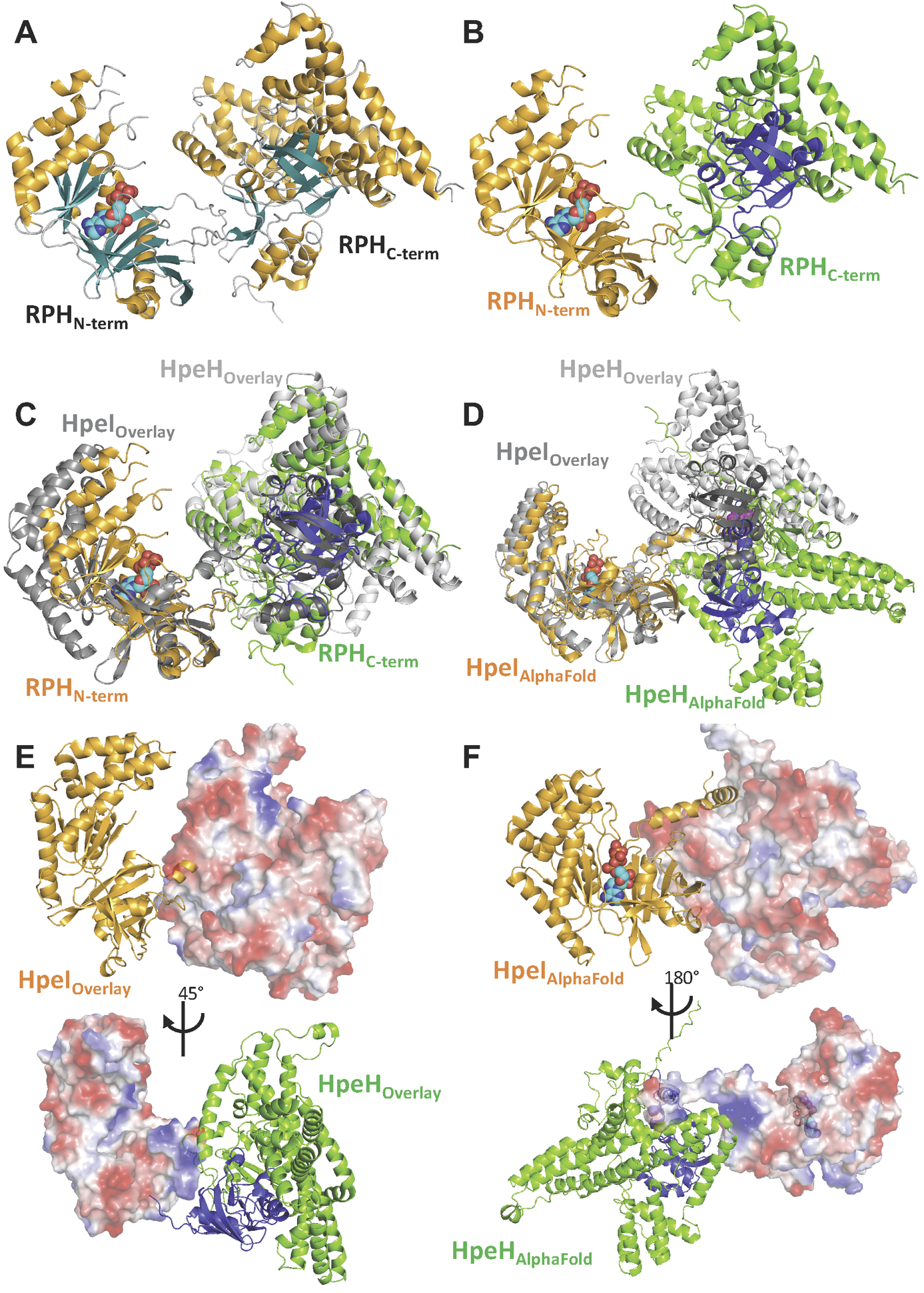
**

**Figure S16. Models of a potential HpeHI complex.** (**A**) The structure of the homologous fusion protein, RPH (PDB ID: 5FBS) shown as cartoons with α-helixes and β-strands colored orange and teal, respectively and bound ADP shown as spheres. (**B**) RPH cartoon colored by functional element with the ATP-grasp, Substrate binding and Swivel domains shown in orange, green and blue, respectively. (**C**) Superposition of the HpeI crystal structure (dark grey) and HpeH AlphaFold model (light grey) onto RPH. (**D**) Superposition of two potential HpeHI complexes, generated by superposition on RPH, as in panel C, and the 1:1:1 AlphaFold model of HpeH:HpeI:ATP (see Fig. 3) is shown as green, orange and blue. (**E**) The RPH-superimposed HpeHI model showing the surface contoured electrostatic potential for HpeI and HpeH. Red, white and blue indicate negative, neutral and positive surface potential. (**F**) The ternary HpeH:HpeI:ATP AlphaFold model showing the surface contoured electrostatic potential, colored as in panel E.

**Tables**

**Table S1:** Extinction coefficients of HAP and PAP at 325 nM for pH 7.5, 8.0 and 8.5.

| **pH** | **7.5** | **8.0** | **8.5** |
| --- | --- | --- | --- |
| **HAP** | 6.3 | 13.1 | 19.6 |
| **PAP** | 0.2 | 0.2 | 0.2 |
| **Δε_325nm_** | 6.2 | 12.9 | 19.5 |

**Table S2:** Phosphate transfer from PAP to AV by HpeH.

| **Reaction Condition** | **Rate of PAV production** |
| --- | --- |
|  | nmol min^-1^ mg^-1^ of HpeH |
| HpeH + PAP | 2.7 ± 0.5 |
| HpeH | 0 |
| HpeH + PAP + AMP-PNP | 3.4 ± 0.4 |
| HpeH + AMP-PNP | 0 |
| HpeH+HpeI + PAP | 2.3 ± 0.2 |
| HpeH+HpeI + PAP + AMP-PNP | 3.5 ± 0.3 |
| HpeH+HpeI + AMP-PNP | 0 |
| PAP | 0 |

**Table S3:** Crystallographic data and refinement statistics.

| **Parameter** |  |
| --- | --- |
| **Data collection** |  |
| Beamline | DLS I04 |
| Wavelength | 0.95374 |
| Space group | *P*2_1_ |
| Cell dimensions a, b, c (˚A) | 45.9, 57.3, 67.1 |
| *α*, *β*, *γ* (°) | 90.0, 99.6, 90.0 |
| Resolution (˚A) | 66.18-1.80 (1.97-1.80)*^a^* |
| *R_merge_* [%] | 16.4 (131.2) |
| *R_pim_* [%] | 6.7 (52.8) |
| ⟨*I/σ*(*I*)⟩ | 6.9 (1.4) |
| Completeness (%) | 90.7 (66.0)*^b^* |
| Redundancy | 7.0 (7.2) |
| CC(1/2) | 0.996 (0.642) |
| **Refinement**  *Rwork* / *Rfree* | 20.7 / 25.5 |
| Ramachandran plot  most favored [%] | 96.9 |
| allowed [%] | 3.1 |
| disallowed [%] | 0.0 |
| No. atoms  protein | 2564 |
| water | 88 |
| B-factors  protein | 24.8 |
| water | 21.0 |
| R.m.s. deviations  Bond lengths (˚A) | 0.0067 |
| Bond angles (°) | 1.78 |
| pdb-code | 9GOJ |

*^a^*values in parentheses are for the highest-resolution shell

*^b^*ellipsoidal completeness

**Table S4**: Primers used in this study.

| Primer | Description | Sequence (5’-3’) |
| --- | --- | --- |
| oGD19/20 | pET28a_*hpeH* | AACCTGTATTTTCAGGGCCATATGACGATCACCGAGAGCAAGC  AGTGGTGGTGGTGGTGGTGCTTAAGTCAGGAGGCGTCAGCGGC |
| oGD35/36 | pGD108 | CATCATGTGCCAGGCCGCGATCG  CCGCCGATATCGCTGACC |
| oGD37/38 | pGD109 | CGGCATCATGAGCCACGCCGCGA  CCGATATCGCTGACCGCTCCG |
| oGD39/38 | pGD110 | CGGCATCATGGCGCACGCCGCGA  CCGATATCGCTGACCGCTCCG |
| oGD44/45 | pET15B_*hpeI* | AACTTGTATTTCCAGGGCCATATGGGTAAGTACACGAAGAAGTTCAAC  TTGACAGCTTATCATCGATAAGCTTTCATCGGGTCTGTCCCTTC |

**Table S5**: Plasmids used in this study.

| Plasmid | Description | Source | |
| --- | --- | --- | --- |
| pET15b | Protein production, empty vector | Novagen |  |
| pET28a | Protein production, empty vector | Novagen |  |
| pGD101 | pET28a derivative with *hpeH* cloned between *Nde*I/XhoI cut sites by Gibson. | This study |  |
| pGD106 | pET15b derivative with *hpeI* cloned between NdeI/HindIII cut sites by Gibson. | This study |  |
| pGD108 | pET28a derivative with *hpeH* H580Q mutant cloned between NdeI/XhoI cut sites by site directed mutagenesis. | This study |  |
| pGD109 | pET28a derivative with *hpeH* C581S mutant cloned between NdeI/XhoI cut sites by site directed mutagenesis. | This study |  |
| pGD110 | pET28a derivative with *hpeH* C581A mutant cloned between NdeI/XhoI cut sites by site directed mutagenesis. | This study |  |
